# Regulation of Chemical Transformation in Designer Peptide Biomolecular Condensates

**DOI:** 10.1021/acsami.5c21674

**Published:** 2026-02-18

**Authors:** Shirel Veretnik, Avigail Baruch Leshem, Ayala Lampel

**Affiliations:** † Shmunis School of Biomedicine and Cancer Research, George S. Wise Faculty of Life Sciences, 26745 Tel Aviv University, Tel Aviv 69978, Israel; § Center for Nanoscience and Nanotechnology, 26745 Tel Aviv University, Tel Aviv 69978, Israel; ∥ Center for the Physics and Chemistry of Living Systems, 26745 Tel Aviv University, Tel Aviv 69978, Israel; ⊥ Leibniz Institute of Polymer Research Dresden, Max Bergmann Center of Biomaterials Dresden, Dresden 01069, Germany

**Keywords:** liquid−liquid phase separation, condensates, peptides, reaction, compartmentalization, CuAAC, click chemistry

## Abstract

Biomolecular condensates, formed through liquid–liquid phase separation, serve as dynamic platforms for biochemical regulation. Inspired by these natural systems, we develop designer peptide-based condensates to modulate chemical transformations, focusing on the Cu­(I)-catalyzed azide–alkyne cycloaddition click reaction between hydrophobic reactants as a model system. By incorporating a varying number of isoleucine residues into peptide sequences, we tune the hydrophobicity of the condensates. This variation allows us to tune condensate properties, including reactant recruitment, internal mobility, and catalytic performance. We show that peptide hydrophobicity dictates selective partitioning of the hydrophobic azide reactant into the dense phase, while increased hydrophobicity reduces internal diffusion. Higher molecular mobility within the condensates correlates with increased reaction rates and product formation, leading to enhanced spatially localized reactivity within the condensates. Together, our findings establish a mechanistic framework linking the peptide sequence, condensate dynamics, and compartmentalized catalysis. This work provides a foundation for using designer condensates as programmable microreactors for sustainable chemistry and biomedical applications.

## Graphical abstract

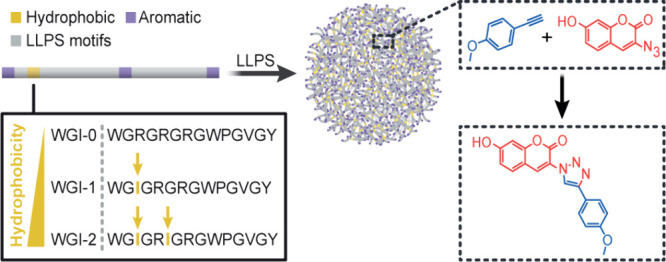

## Introduction

The compartmentalization of biochemical reactions within spatially distinct cellular environments is a hallmark of life, allowing for precise spatiotemporal regulation of complex processes.^1^ For example, ATP synthesis occurs within mitochondria, leveraging the proton gradient to drive energy production, while proteolysis is compartmentalized in lysosomes, which isolates degradative enzymes to ensure cellular homeostasis. Beyond membrane-bound organelles, many cellular functions are orchestrated within membraneless organelles, or biomolecular condensates,^2^ which are formed through liquid–liquid phase separation (LLPS) and provide dynamic, compartmentalized environments for biochemical transformations. Such condensates, including nucleoli and nuclear stress bodies, create specialized microenvironments for processes like ribosome assembly^3^ and phosphorylation of splicing factors,^4^ respectively.

The physical basis of LLPS lies in networks of weak, noncovalent interactions among intrinsically disordered proteins (IDPs), or peptides,^5–8^ either in the absence or presence of nucleic acids,^2^ including electrostatic interactions, hydrogen bonds, and π–π stacking.^9,10^ This interplay of interactions generates dense, liquid-like phases with unique physicochemical properties,^11^ which enable the selective recruitment, sequestration, and transformation of biomolecules.^12,13^ These features have inspired the design of synthetic biomolecular condensates for diverse applications, including as enzyme microreactors, molecular delivery systems,^14^ and platforms for catalytic transformations^15^ including compartmentalization of organic reactions such as aldol and hydrazone formation,^16^ redox reactions,^17,18^ imine synthesis,^19^ light-triggered radical polymerization,^20^ retro-Diels–Alder reaction,^21^ and selective amide bond formation.^22^

Despite the potential of synthetic condensates, understanding the determinants that govern their performance as reaction vessels—particularly how dense-phase chemical properties affect reactant recruitment, reaction efficiency, and product localization—remains limited. Of particular interest is the role of hydrophobic microenvironments, which can recruit hydrophobic reactants and mediate reactions in aqueous systems, avoiding the need for organic solvents. This approach aligns with principles of green chemistry by reducing the environmental impact while enhancing the reaction efficiency.

Here, we present a systematic exploration of hydrophobicity as a design parameter in synthetic peptide-based condensates. Employing chemically diverse building blocks such as peptides^23–26^ enables the engineering of condensates with a rich set of chemical properties.^7,27,28^ Using minimalistic peptides, we engineered condensates with tunable hydrophobicity by incorporating varying numbers of isoleucine residues into the peptide sequence. As a model system, we employed the Cu­(I)-catalyzed azide–alkyne cycloaddition (CuAAC), i.e., click chemistry reaction,^29^ a widely used transformation in drug discovery, proteomics, and materials science.^30,31^ This reaction, between two hydrophobic azide–alkyne reactants, forms a traceable aromatic triazole product under mild conditions in aqueous environments, providing an ideal platform for studying the interplay of the condensate microenvironment and reaction kinetics.

Our findings demonstrate that peptide-based condensates significantly enhance the rate and yield of CuAAC reactions compared to bulk solution. By systematically tuning peptide hydrophobicity, we reveal its dual role in modulating both the phase separation propensity and internal condensate dynamics. While moderate hydrophobicity supports optimal reactivity through fluid and dynamic condensates, excessive hydrophobicity leads to reduced molecular mobility and aggregation. Furthermore, we show that Cu^2^^+^ ions can be removed after reaction completion through simple centrifugation, facilitating downstream processing. These findings highlight the importance of balancing condensate fluidity and composition to engineer biomolecular condensates as tunable, sustainable microreactors to enable precise control over reaction localization, rate, and product formation for applications in green chemistry.

## Results and Discussion

### Design of Peptide Condensates with Varying Hydrophobicity

We aimed to develop biomolecular condensates that function as reaction vessels and facilitate spatial modulation of chemical transformations. Building on our prior work, we utilized a minimalistic 14-mer peptide designed to promote LLPS via simple coacervation, forming biomolecular condensates.^7^ The peptide sequence comprises three repeats of glycine-arginine (Gly-Arg) dyads, which are common in low-complexity domains of IDPs and promote phase separation by providing backbone flexibility. Additionally, the peptide includes three aromatic amino acids—two tryptophans (Trp) and one tyrosine (Tyr)—which drive π–π interactions among themselves and with the Arg side chains.^7^ To further enhance LLPS, the peptide incorporates an elastin-like polypeptide domain (Pro-Gly-Val-Gly), known to promote phase separation through simple coacervation.^32,33^ To investigate how condensate properties, particularly hydrophobicity, influence reaction efficiency in terms of rate and product formation, we designed a small library of peptide building blocks with varying hydrophobicity by introducing different numbers of the aliphatic amino acid isoleucine (Ile). We selected Ile owing to the long-branched aliphatic characteristic of its side chain, which we expected to facilitate the recruitment of hydrophobic reactants to the dense phase of the condensates and, thus, increase reaction efficiency. The primary peptide, lacking Ile, was named WGI-0. To introduce a single Ile residue while preserving the sticker–spacer design,^34^ we replaced the Arg at position 3 with Ile, generating WGI-1. A second Ile was incorporated by substituting the Gly at position 6, yielding WGI-2 (Figure 1a and Figure S1). The calculated hydrophilicity values of WGI-0, WGI-1, and WGI-2 are −0.11, −0.46, and −0.59, respectively. In addition, the HPLC chromatograms of the peptides indicate that WGI-2 is the most hydrophobic peptide and that WGI-0 is the least hydrophobic peptide, as reflected by their retention time (Figure S1).

**Figure 1:**
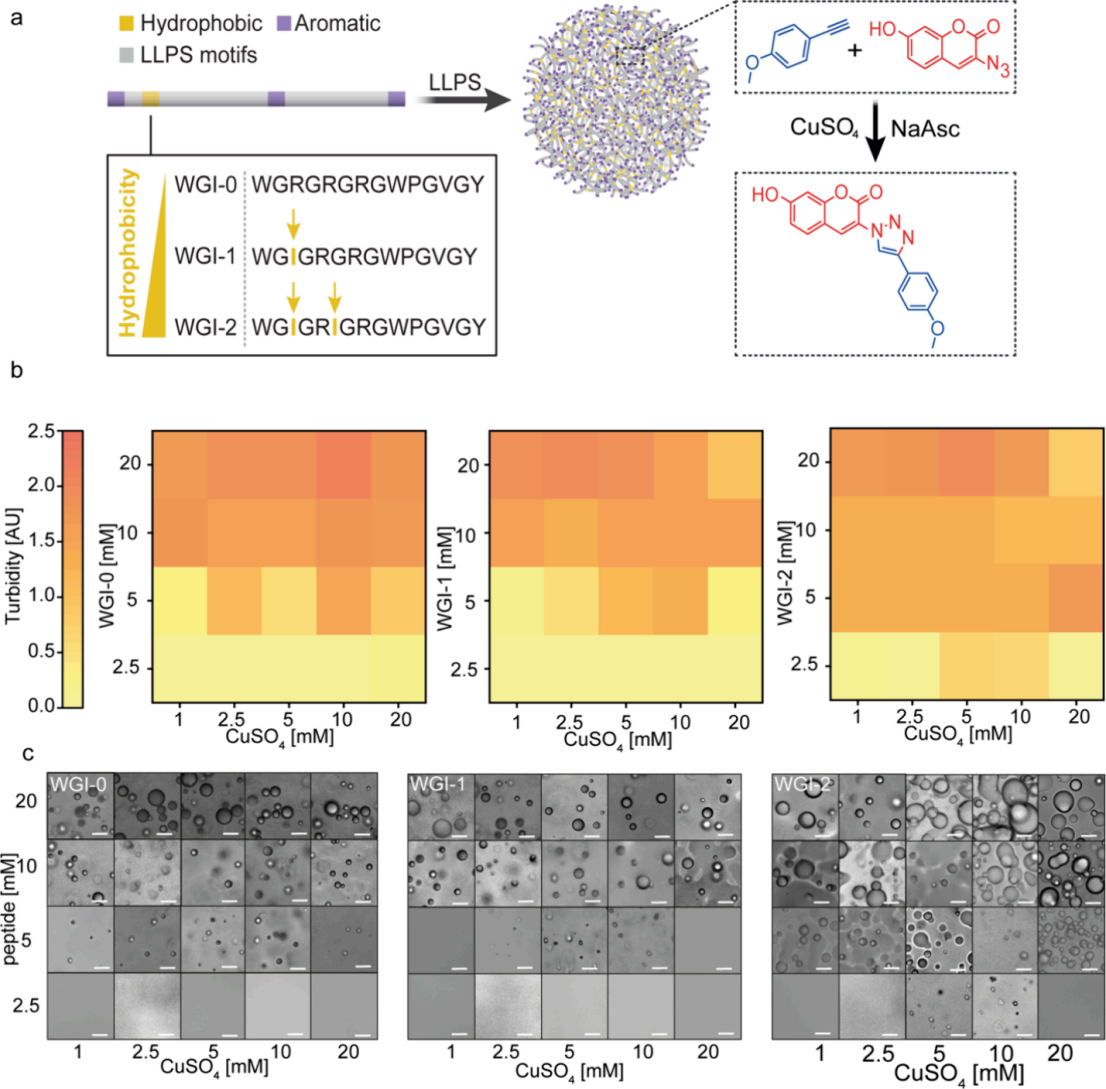
Hydrophobicity of peptide condensates regulates chemical reactions. (a) Schematics of the suggested system: LLPS-promoting peptide building blocks with varying levels of hydrophobicity form biomolecular condensates for regulation of a click chemistry reaction. (b) Phase diagram heatmaps of WGI-0, WGI-1, and WGI-2 as a function of peptide and CuSO_4_ concentration. Peptides were dissolved in 20 mM citrate buffer, pH 5.4. Turbidity intensity was monitored at λ = 400 nm. (c) Microscopy analysis of the peptides as a function of peptide and CuSO_4_ concentration. Scale bar = 10 μm.

As a model reaction system, we focused on the Cu­(I)-catalyzed cycloaddition reaction between azide and terminal alkyne (CuAAC) to form a 1,2,3-triazole (Figure 1a and Figure S2). Click reactions are well-characterized and widely used in drug discovery and proteomic studies,^29^ making them an ideal model for studying compartmentalized drug synthesis. Since the reaction requires Cu­(I) as a catalyst,^35^ we employed CuSO_4_, which acts both as a catalyst and an initiator for LLPS by screening the charges of the cationic peptides, thereby promoting intermolecular interactions and simple coacervation. We first evaluated the LLPS propensity of each peptide as a function of the peptide and CuSO_4_ concentration. Peptides were dissolved in citrate buffer at pH 6, which decreases to 5.4 after dissolution and CuSO_4_ addition, at final concentrations of 2.5, 5, 10, and 20 mM. Phase diagrams were constructed by measuring sample turbidity (λ = 400 nm), following addition of CuSO_4_ at varying concentrations of 1–20 mM. At this wavelength, the intensity reflects light scattering by condensates that corresponds to turbidity rather than peptide absorbance (Figure 1b and Figure S3). Microscopy analysis confirmed that the turbidity resulted from condensate formation rather than aggregate formation (Figure 1c). For WGI-0 and WGI-1, the addition of CuSO_4_ triggers phase separation and condensate formation (Figure S4a,b). In contrast, WGI-2, above 5 mM, undergoes LLPS immediately when dissolved in buffer (Figure S4c). This indicates that the higher hydrophobicity of WGI-2 promotes attractive forces that overcome the electrostatic repulsion between the peptide molecules, resulting in LLPS and condensate formation.

The turbidity and microscopy analyses revealed that the LLPS propensity of all three peptides was concentration-dependent, with turbidity increasing as a function of peptide concentration. WGI-0 exhibits a slightly higher LLPS propensity than WGI-1. At both the lowest and highest CuSO_4_ concentrations (1 and 20 mM), WGI-0 shows clear turbidity and droplet formation, while WGI-1 does not undergo LLPS under the same conditions (Figure 1b,c). These results support the conclusion that WGI-0 phase separates more readily than WGI-1. The most hydrophobic peptide, WGI-2, showed the strongest LLPS propensity, reflected by its lowest saturation concentration (*C*_sat_) of 2.5 mM. These findings show that phase separation propensity does not follow a simple monotonic trend based solely on hydrophobicity. Instead, LLPS emerges from a balance between the overall hydrophobic character of the peptide and the nature of the specific interaction motifs within the sequence. Thus, replacing Arg with Ile in WGI-1 increases the hydrophobicity but simultaneously removes a highly effective sticker residue, resulting in reduced LLPS despite the increased hydrophobic content. In contrast, WGI-2 compensates for the loss of Arg by incorporating two Ile residues, yielding a peptide whose enhanced hydrophobicity exceeds the reduction in sticker strength and ultimately promotes phase separation more strongly.

### Regulation of Reactant Recruitment to the Condensates

To investigate how the chemical environment of peptide condensates modulates reactant recruitment, we selected the CuAAC reaction between 3-azido-7-hydroxycoumarin (AHC) and 4-ethynylanisole as a model system. This transformation results in a fluorescent product (λ_ex_ = 330 nm, λ_em_ = 460, Figure S5). We first evaluated the encapsulation efficiency (EE) of AHC in the condensate dense phase by analyzing its concentration in supernatants following centrifugation, using HPLC, calibrated against standard AHC curves (Figure S6). AHC was initially dissolved in 100% tert-butanol (t-BtOH) to generate a stock solution and then diluted into the LLPS samples or buffered to a final t-BtOH concentration of 5%. We verified that 5% t-BtOH does not affect condensate formation or morphology, using bright-field microscopy (Figure S7). In citrate buffer (pH 5.4), condensates formed by 5 mM of WGI-0, WGI-1, and WGI-2 had relatively low EE of 7.2 ± 5.5%, 35.8 ± 3.1%, and 27.7 ± 1.7, respectively (Figure 2a,b, Figure S8a, and Table S1). Increasing the peptide concentration to 10 and 20 mM further enhanced AHC recruitment, yielding EE values of up to 43.0 ± 6.7% (WGI-0), 65.1 ± 7.5% (WGI-1), and 69.4 ± 9.7 (WGI-2) (Figure 2a,b and Figure S8a). Replacing citrate with MES buffer (pH 5.7), which binds Cu­(II/I) more weakly, enhanced the EE in both WGI-0 and WGI-1 condensates. At a peptide concentration of 20 mM, EE reached its highest levels, with 63.7 ± 2.6% for WGI-0 and 91.6 ± 1.6% for WGI-1 (Figure 2c,d and Table S1). Quantification of dense-phase peptide concentration for WGI-0 in MES buffer (Figure S9) revealed a clear correlation between dense-phase peptide content and EE across varying total peptide concentrations, indicating that the increased peptide density at 20 mM facilitates more efficient AHC recruitment.

**Figure 2:**
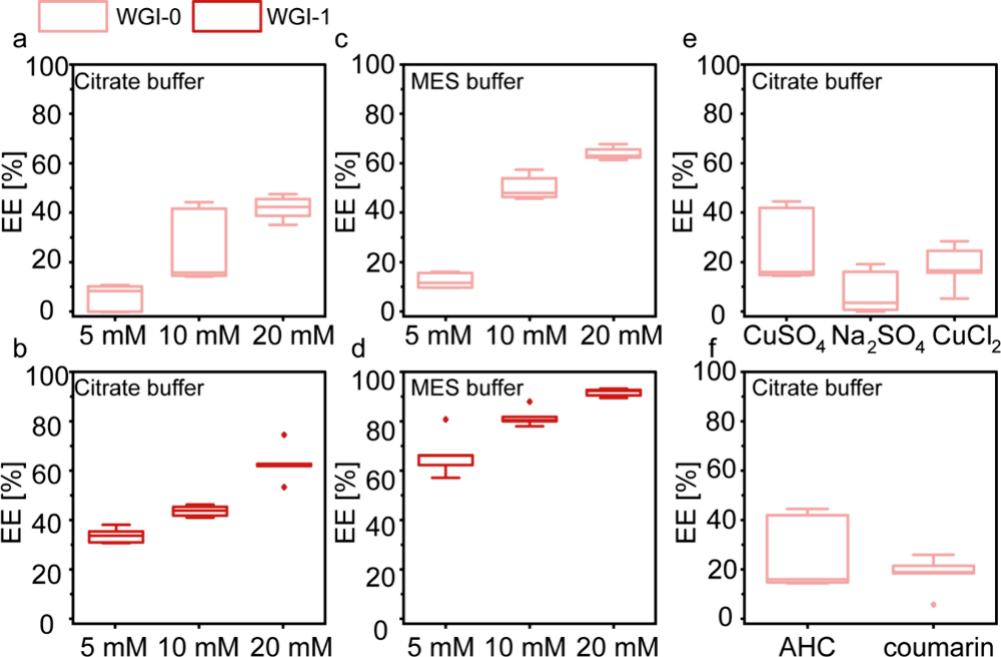
Reactant recruitment to peptide condensates. Encapsulation efficiency (EE) analysis of the reactant AHC to WGI-0 and WGI-1 condensates prepared in citrate (a–b, e–f) or MES (c, d) buffer. (a, b) EE of AHC in WGI-0 (a) and WGI-1 (b) condensates in citrate buffer, pH 5.4, at varying peptide concentrations. (c, d) EE of AHC in WGI-0 (c) and WGI-1 (d) condensates in MES buffer, pH 5.7, at varying peptide concentrations. (e) EE of AHC in WGI-0 condensates in citrate buffer with varying salts. (f) EE of AHC vs coumarin in WGI-0 condensates prepared in citrate buffer. Values are presented as box plots based on *n* = 4–6 independent experiments.

To determine whether Cu^2^^+^ ions limit reactant recruitment, we replaced CuSO_4_ with Na_2_SO_4_ or CuCl_2_ in the WGI-0 condensates. These substitutions yielded EE values of 7.9 ± 9.0% and 18.1 ± 9.0%, respectively, indicating that the counterions do not significantly restrict encapsulation (Figure 2e). We also tested whether electrostatic interactions between the cationic peptides and the azido group of the AHC influence recruitment (Figure 2f). Surprisingly, the neutral compound coumarin, structurally similar but lacking the azide moiety, exhibited even lower EE (18.1 ± 7.5 compared to 26.3 ± 15.5), suggesting that electrostatic repulsion does not account for the differences observed.

### Preparation of Condensates for Kinetic Studies and Reaction Monitoring

Next, we analyze the impact of peptide condensates on reaction kinetics by monitoring the concentration of the triazole product 7-hydroxy-3-(4-(4-methoxyphenyl)-1*H*-1,2,3-triazol-1-yl)-2*H*-chromen-2-one (Figure S10 shows product verification) at specific time points using HPLC. The reaction involves the reduction of Cu­(II) to Cu­(I) by sodium ascorbate (NaAsc), which activates the ethynyl group, allowing azide to form the triazole ring. To minimize Cu­(I) oxidation, all reactions were carried out under an argon (Ar) atmosphere. Reactants were used at a final concentration of 0.5 mM, as higher concentrations destabilize the condensates during the reaction (Figure S11). Peptides (5 mM) were dissolved in citrate buffer (5 mM peptide, pH 5.4) or MES buffer (20 mM peptide, pH 5.7), followed by addition of NaAsc and reactants, and dissolved in t-BtOH (5% v/v final concentration), with the subsequent 30 min incubation under Ar (degassing) and thereafter addition of CuSO_4_ (10 mM). For the reaction in citrate buffer, the salt addition triggered both droplet formation and reaction initiation (Figure 3a, left panel), and for the reaction in MES buffer at this higher peptide concentration, LLPS is triggered after pH adjustment and before CuSO_4_ addition (Figure 3a, right panel, Figure S12). The most hydrophobic peptide, WGI-2, formed droplets before CuSO_4_ addition (Figure S3).

**Figure 3:**
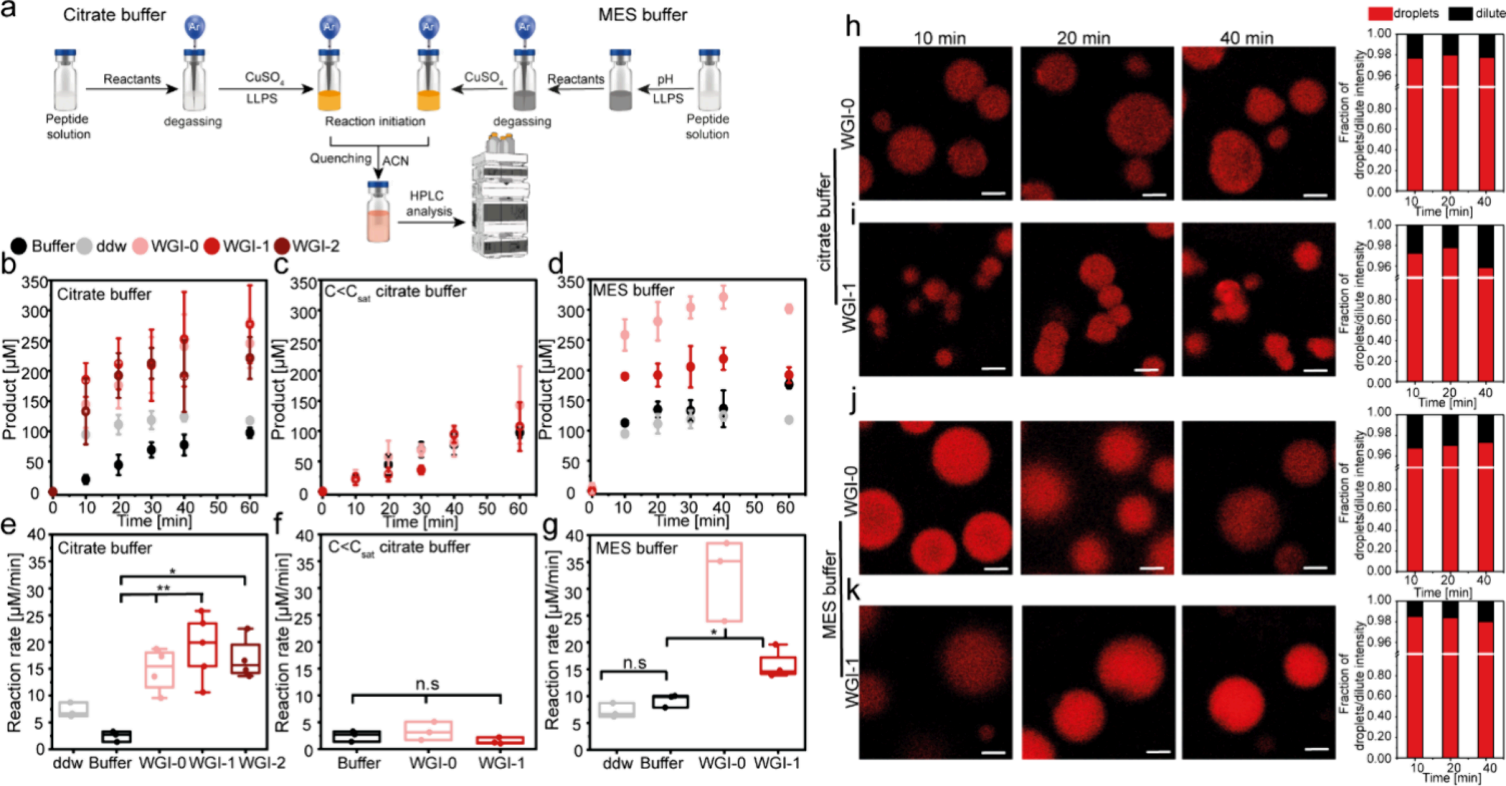
Reaction kinetics in phase-separated systems. (a) Schematics of sample preparation of the reactions in peptide condensates, either in citrate or MES buffer and their analysis using HPLC. (b, c) Reaction kinetics in citrate buffer, pH 5.4, in (b) phase-separated systems (5 mM peptide) and (c) in the presence of WGI-0 or WGI-1 at a concentration below *C*_sat_ (2 mM), where no condensates form. (d) Reaction kinetics in phase-separated systems in MES buffer (20 mM peptide, pH 5.7). (e–g) Corresponding reaction rates in (e, f) citrate buffer and (g) MES buffer. All reactions contain 5% of t-BtOH. Values represent averages (b–d) or box plots (e–g) of *n* = 3–4 independent analyses for each system. Error bars in (b–d) represent SD. *p value <0.05, **p value <0.01. (h–k) CLSM analysis of product fluorescence in WGI-0 and WGI-1 phase-separated systems in (h, i) citrate and (j, k) MES buffer. Images collected using λ_ex_ = 405 nm. Scale bars = 2 μm. The relative fluorescence intensity of products in the dense vs dilute phase obtained from CLSM analysis is presented on the right panel. Values in each bar represent *n* = 43–110 droplets/dilute phase.

Reaction kinetics was monitored using HPLC by measuring the product concentration over time. For this, the reaction was quenched at specific time points by addition of acetonitrile (ACN) at a final volume percentage of 50% v/v immediately before injection to the HPLC (Figure 3a). Thus, this analysis represents product formation in the overall heterogeneous mixture of both the dilute and dense phases. Product concentration over time was calculated based on the product calibration curve (Figure S6a, see the methods section).

### Phase-Separating Peptide Systems Enhance Reaction Kinetics

The reactions in all peptide condensates were significantly more efficient than those in citrate buffer or in ddw, in terms of both the product concentration and reaction rate (Figure 3b,e). The reaction rate was 6-fold faster for WGI-0, ∼8-fold for WGI-1, and ∼7-fold for WGI-2, than that in bulk buffer, where no significant difference in rate was found between the peptides. Yet, bright-field microscopy showed that while WGI-0 and WGI-1 condensates remained stable throughout the reaction, WGI-2 condensates aggregated over reaction time (Figure S13a). Further analysis revealed that WGI-2 aggregation is a result of product formation since no aggregation was observed with NaAsc or the reactants alone (Figure S14). Transmission electron microscopy (TEM) analysis of WGI-2 condensates at *t* = 40 min of the reaction showed abundant aggregation around the remaining droplets (Figure S15). This aggregation might be a result of droplet destabilization by product accumulation and can also be facilitated by the strong kosmotrope sulfate (SO_4_^2–^) ions through water exclusion and hydrophobic interactions of the hydrophobic peptide and product.

When reactions were conducted below the *C*_sat_, where no condensates form, the product concentration and reaction rate for WGI-0 and WGI-1 peptides were similar to those in buffer, highlighting the role of the dense phase in enhancing reaction efficiency (Figure 3c,f). The slow reaction rate in citrate buffer suggests that citric acid ions inhibit the reaction, possibly by acting as ligands to Cu­(II)/Cu­(I) and competing with its interaction with the ethynyl group. Since citrate buffer facilitates phase separation through charge screening,^20^ it is plausible that interactions between the cationic peptides and citric acid compete with its binding to Cu­(II)/Cu­(I). This interaction may mitigate the inhibitory effect of citric acid ions, resulting in higher conversions in these systems. Performing the reactions in condensates formed in MES buffer at a higher peptide concentration (20 mM) increased the reaction rate by 3.5-fold and 2-fold in WGI-0 and WGI-1, respectively, compared to bulk buffer solution and 4.5-fold and 2-fold by WGI-0 and WGI-1, respectively, compared to ddw (Figure 3d,g). Similarly, the condensates exhibited a faster coalescence compared to those in citrate buffer at a lower peptide concentration (Figure S13).

To further understand the spatial dynamics of product formation within phase-separated systems, we performed CLSM analysis to visualize the fluorescence of the triazole product throughout the reaction course. Continuous real-time CLSM imaging led to markedly slower kinetics (Figure S16) due to oxidation of Cu­(I) by air. Therefore, reactions were conducted under an Ar atmosphere and sampled at defined time points (*t* = 10, 20, and 40 min) for end point imaging. CLSM imaging revealed that for all peptide systems in both citrate and MES buffers, the fluorescent product accumulated preferentially within the condensates, with over 90% of the total signal localized in the dense phase (Figure 3h–k).

### The Diffusion in the Condensates Correlates with Reaction Efficiency

To assess whether the material properties of the condensates influence the observed reaction rates, we performed fluorescence recovery after photobleaching (FRAP) analysis of rhodamine B-loaded condensates formed in citrate buffer using CLSM. Diffusion of rhodamine B within the dense phase provides insight into condensate dynamics. We first determined the EE of rhodamine B in WGI-0, WGI-1, and WGI-2 condensates, prepared at a 10 mM peptide concentration, as the 5 mM peptide yielded condensates that were too small or dynamic for reliable CLSM analysis. EE values, obtained by absorbance spectroscopy of the dilute phase following condensate centrifugation and using calibrated rhodamine B (Figure S6d), were 97%, 90%, and 94% for WGI-0, WGI-1, and WGI-2, respectively (Figure 4a). For WGI-2, which also forms condensates without CuSO_4_, the EE in the absence of CuSO_4_ was 99%, indicating that CuSO_4_ slightly restricts rhodamine B recruitment.

**Figure 4:**
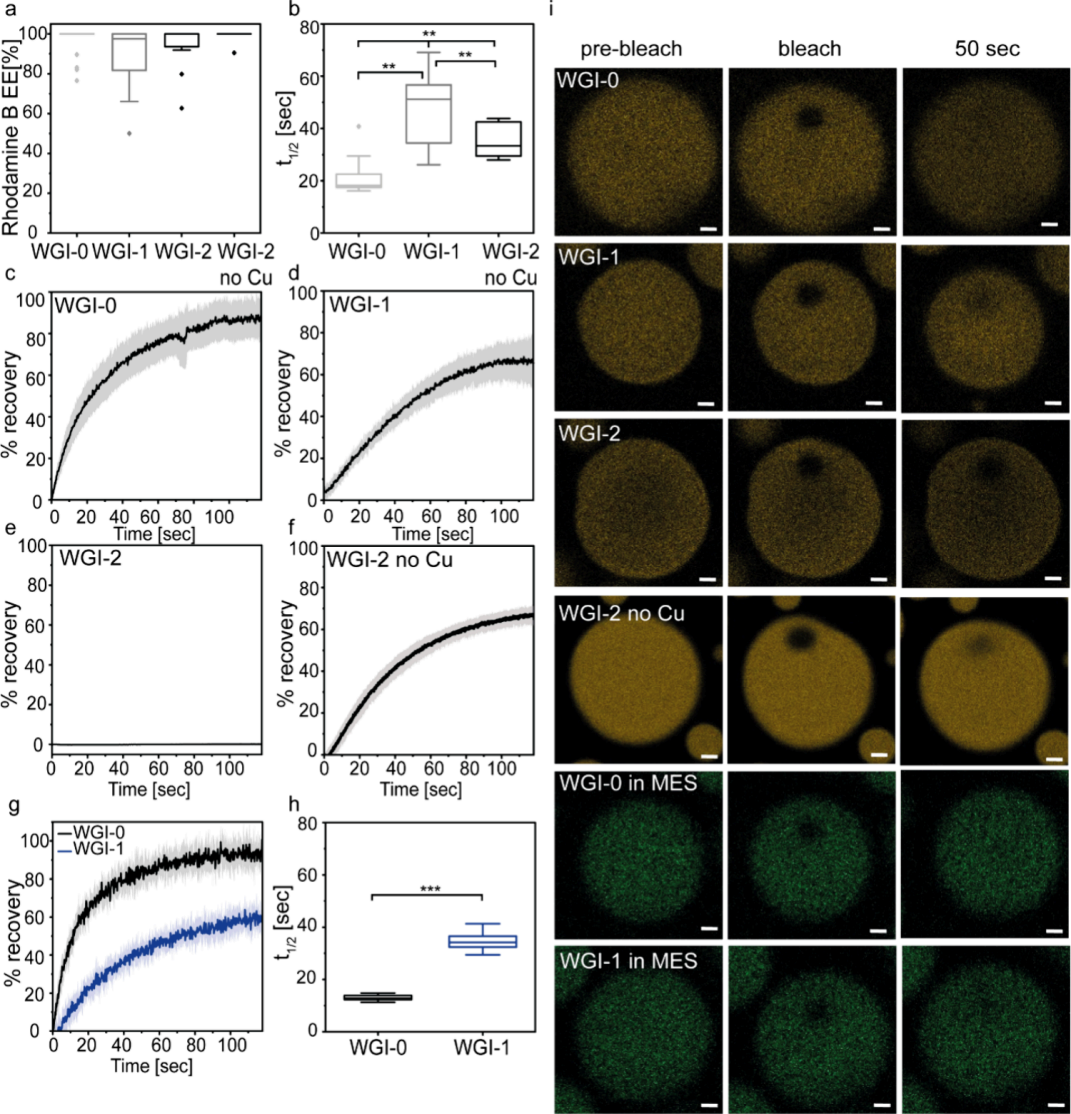
Condensate dynamics is directly affected by the number of Ile in the peptide sequence. (a) Encapsulation efficiency (EE) of rhodamine B in WGI-0, WGI-1, and WGI-2 condensates and in the WGI-2 condensate without CuSO_4_. (b) *t*_1/2_ values of WGI-0, WGI-1, and WGI-2 condensates (10 mM in citrate buffer, pH 5.4) without CuSO_4_. (c–f) Recovery plots of (c) WGI-0, (d) WGI-1, (e) WGI-2, and (f) WGI-2 condensates without CuSO_4_. Recovery plots represent average or box plots based on *n* = 11 condensates for WGI-0, *n* = 17 for WGI-1, *n* = 8 for WGI-2, and *n* = 9 for WGI-2 without CuSO_4_. (g, h) Recovery plots (g) and the respective *t*_1/2_ values (h) of WGI-0 and WGI-1 (20 mM) in MES buffer, pH 5.7. (i) CLSM micrographs of FRAP analysis before, immediately after, and 50 s after photobleaching. FRAP analysis was performed using rhodamine B (10 μM) and fluorescein (10 μM) in citrate and MES buffer, respectively. Scale bars = 2 μm. **p value <0.01, ***p value <0.001.

FRAP analysis revealed a marked effect of peptide hydrophobicity on condensate dynamics (Figure 4b–f, i). Increasing the number of Ile residues from zero to one increased the recovery half-time by 2.3-fold (21.5 ± 7.4 s for WGI-0 vs 48.0 ± 13.3 s for WGI-1). Incorporating two Ile residues (WGI-2) drastically altered material properties, as no recovery of rhodamine B fluorescent signal was detected in the presence of CuSO_4_ (Figure 4e). Thus, although the recruitment of rhodamine B to the dense phase is highly efficient (94%), its internal mobility in the dense phase is arrested. Moreover, while no fluidity is observed in WGI-2 condensates, the AHC reactant is homogeneously distributed in the dense phase (Figure S8b,c). In the absence of CuSO_4_, however, WGI-2 condensates recovered with *t*_1/2_ = 35.8 ± 6.7 s, suggesting that CuSO_4_ attenuates diffusion. Notably, the presence of the salt seems to decrease the fluorescence intensity (Figure 4i). The reduced dynamics of WGI-2 condensates are consistent with their propensity to aggregate during the reaction course.

Considering that AHC recruitment was low for all three systems in citrate buffer (Figure 2a,b), the reaction under these conditions likely occurs predominantly in the dilute phase, making it improbable that diffusion within condensates affects the rate. However, in MES buffer at a higher peptide concentration (20 mM), AHC partitions mainly into the dense phase (Figure 2c,d), suggesting that the reaction occurs in the dense phase and pointing to a direct relationship between diffusion and reaction rate. Hence, we analyzed the diffusion of fluorescein in WGI-0 and WGI-1 condensates (20 mM) in MES buffer. The 2.3-fold faster reaction rate observed in WGI-0 compared to WGI-1 condensates (Figure 3g) is consistent with the ∼2.6-fold slower diffusion (*t*_1/2_) and 1.5-fold lower total recovery in WGI-1 condensates (Figure 4g,h).

### Cu^2+^ Localization in Peptide Condensates

To investigate the localization of Cu^2^^+^ ions within the peptide condensates, we performed scanning transmission electron microscopy (STEM) coupled with energy-dispersive spectroscopy (EDS) on WGI-0, WGI-1, and WGI-2 samples in citrate buffer. High-angle annular dark-field (HAADF) imaging combined with EDS line profiling revealed the presence of Cu^2^^+^ ions within the dense phase of WGI-0 (Figure 5a,b), WGI-1 (Figure 5c,d), and WGI-2 (Figure 5e,f and Figure S17) condensates. Quantitative comparison of the net Cu signal showed that the highest level of accumulation of Cu^2^^+^ occurs in the most hydrophobic condensates formed by WGI-2. This enhanced Cu^2^^+^ retention correlates with the markedly reduced internal mobility observed for WGI-2 condensates in the FRAP analysis. We attribute the enhanced Cu^2^^+^ accumulation in WGI-2 primarily to multivalent weak coordination within the dense phase rather than hydrophobic partitioning. Such interactions are likely mediated by backbone carbonyls, the terminal carboxylate, and Arg side chains. Similar accumulation of divalent metal catalysts within condensates has been reported previously,^36^ including Cu^2+^ in peptide-based condensates.^13^

**Figure 5:**
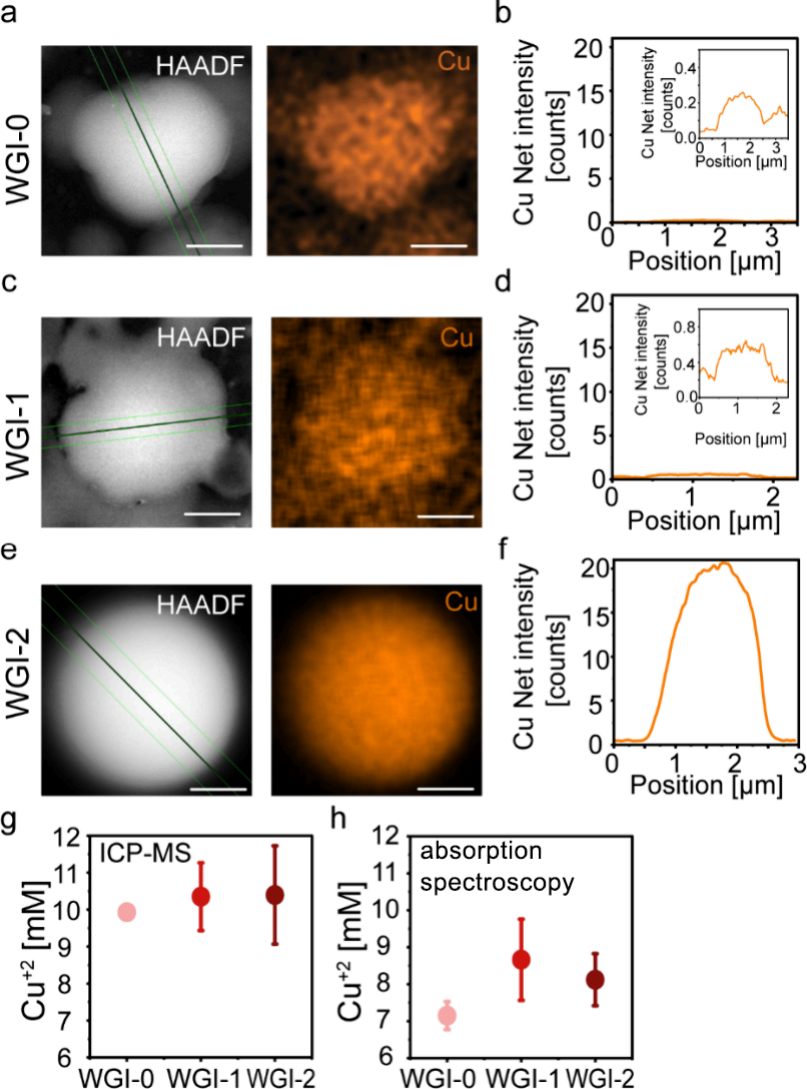
Cu^2+^-ion localization and exclusion from peptide condensates. Condensates were formed by 5 mM peptide and 10 mM CuSO_4_ in citrate buffer, pH 5.4. (a–d) Scanning transmission electron microscopy (STEM) energy-dispersive spectroscopy (EDS) analysis of (a, b) WGI-0, (c, d) WGI-1, and (e, f) WGI-2 condensates. Micrographs showing dark-field and EDS elemental maps of copper (Cu) (a, c, e). Line scan mapping showing the net intensity of Cu (b, d, f). Scale bar = 500 nm. (g, h) Quantification of Cu^2+^ ions in supernatants (dilute phase) following droplet centrifugation using (g) ICP-MS and (h) absorption spectroscopy at λ = 750 nm.

Given that Cu^2^^+^-associated cytotoxicity remains a key barrier for the clinical adoption of CuAAC-based approaches, we next asked whether Cu^2^^+^ ions could be effectively removed by simple postcondensation processing. To this end, we quantified the concentration of Cu^2^^+^ ions remaining in the supernatant after centrifugation of the condensates, using both inductively coupled plasma–mass spectrometry (ICP-MS) and absorption spectroscopy (λ = 750 nm). In both methods, quantification was based on standard calibration curves for Cu^2^^+^ (Figure S18). ICP-MS analysis indicated complete exclusion of Cu^2^^+^ ions from the condensate pellet (Figure 5g), while absorption spectroscopy showed that approximately 71%, 86%, and 81% of the Cu^2^^+^ ions were removed from the supernatant following centrifugation of WGI-0, WGI-1, and WGI-2 condensates, respectively. These results suggest that Cu^2^^+^ sequestration within the condensates is reversible and that a significant fraction can be removed through straightforward processing steps, potentially reducing toxicity concerns in downstream applications.

## Conclusions

In this work, we show that the distinctive chemical environment formed within designed peptide-based biomolecular condensates directly regulates the reaction rate and product formation. By systematically varying the number of aliphatic Ile residues in the peptide sequence, we tuned the hydrophobicity of the condensate dense phase and, consequently, its material and functional properties. Importantly, our results show that peptide hydrophobicity alone does not dictate phase separation propensity; rather, LLPS emerges from a balance between sticker identity and side-chain hydrophobicity, highlighting the critical role of sequence design.

Beyond hydrophobic recruitment, which has previously been shown to enhance partitioning of hydrophobic reactants and catalysts into condensates and thereby increase reaction rates,^13,36^ our findings reveal that internal diffusivity within the dense phase is a key determinant of CuAAC reaction efficiency. Higher molecular mobility within the condensates correlates with increased reaction rates and product formation. This relationship becomes apparent only when the reaction predominantly occurs within the dense phases, as dictated by effective reactant recruitment.

Finally, we show that Cu^2+^ ions accumulate within the condensate dense phase during catalysis but can be efficiently excluded following centrifugation, enabling their removal in downstream processing. This property positions peptide condensates as promising and safer microreactors for CuAAC, particularly for applications involving the production of therapeutic products when residual Cu^2+^ toxicity is a major concern.

Altogether, these results demonstrate that balancing microenvironment hydrophobicity and sticker composition by careful sequence design provides a means for regulating diffusivity, recruitment of hydrophobic reactants, and reaction rate. More broadly, this work shows the potential of designer peptide condensates as microreactors for enhancing the efficiency of reactions in water, offering a sustainable alternative to organic solvents.

## Experimental Section

### Materials and Reagents

Peptides were custom-synthesized and purified with GenScript (Hong Kong). 4-Ethynylanisole, rhodamine B, t-BtOH, and sodium sulfate (NaSO_4_) were manufactured by Acros Organics; AHC by Apollo Scientific, copper sulfate (CuSO_4_) by Fisher Bioreagents; sodium ascorbate (NaAsc) by Glentham Life Sciences; copper­(II) chloride dihydrate (CuCl_2_·2H_2_O) by Combi Blocks; 7-hydroxycoumarin (coumarin) by Aaron Chemical; acetonitrile (ACN) by Biolab. Buffer salts: sodium citrate by Chem-Impex International, citric acid by Alfa Aesar, and MES by Glentham Life Sciences. The reagents for the click reaction, AHC and 4-ethynylanisole, are not soluble in water.

### Peptide Solution Preparation

All peptide condensates were prepared as follows: The desired peptide was dissolved in 20 mM citrate or MES buffer at final pH of 5.4 or 5.7, respectively, at final concentrations of 5, 10, or 20 mM. The calculated hydrophilicity values of WGI-0, WGI-1, and WGI-2 were obtained from the Bachem Peptide calculator (https://www.bachem.com/knowledge-center/peptide-calculator/).

### Phase Diagrams

Condensates were prepared by mixing a stock solution of peptides (final concentrations of 2.5, 5, 10, and 20 mM) dissolved in 20 mM citrate buffer, pH 6, with 1% (v/v%), except for 20 mM CuSO_4_, which dissolved in ddw (solution B) to final concentrations of 1, 2.5, 5, 10, and 20 mM. Condensates were formed immediately upon mixing. All measurements were performed at room temperature. Turbidity was measured in triplicate by an optical density at λ = 400 nm in a BioTek H1 synergy plate reader using a 384-well plate. Bright-field imaging was taken for samples at reported concentrations by a fluorescence microscope (Olympus IX83) and a ×40/0.95 NA Universal Plan Extended Apochromat objective. Images were collected and processed by using CellSens Dimension software.

### Encapsulation Efficiency (EE) Analysis of AHC and Coumarin Using HPLC

To analyze the EE of the AHC reactant or coumarin in the peptide condensates, condensates were prepared by dissolving the peptides as described above at either 5, 10, or 20 mM in the indicated buffer (either citrate or MES buffer) and subsequently adding 10 mM AHC/coumarin to peptide solutions. Droplets were formed by subsequent addition of 10 mM CuSO_4_/NaSO_4_/CuCl_2_ (stock solution at 1M). Then, the droplet solutions were centrifuged at 20 °C, at 17K rcf for 30 min. For HPLC analysis of the payload in the dilute phase, 100 μL from the supernatant was diluted with 100 μL of ACN. AHC or coumarin was monitored by HPLC as detailed in the Preparation of Reactions in Condensates and HPLC Analysis of Reaction Kinetics section, using the gradient 35–40% mobile phase B for 24 min at a flow rate of 1 mL/min.

To quantify the payload concentration, we modeled calibration curves of each payload. For this, AHC was dissolved in a stock solution of 20 mM in t-BtOH. From this solution, serial dilutions were prepared to final concentrations of 0.1, 0.2, 0.3, 0.4, 0.5, 0.6, 0.8, and 1 mM. Coumarin was dissolved in t-BtOH to final concentrations of 0.1, 0.2, 0.3, 0.4, 0.5, and 1 mM. The t-BtOH concentration was kept at 5% v/v. Following addition of 100 μL of ACN, AHC or coumarin solutions at varying concentrations were monitored by HPLC, as detailed in the previous paragraph, using the gradient 35–40% mobile phase B for 24 min at a flow rate of 1 mL/min. The area of the peak was analyzed at an absorption of 214 nm; the retention times of AHC and coumarin were 11.7 and 8 min, respectively. The concentration of either AHC or coumarin in supernatants was calculated by using calibration curves. The %EE values were obtained using the following eq 1:

$$\left(\frac{C_{i}-C_{\sup}}{C_{i}}\right)\times 100$$ 1

where *C*_i_ represents the initial (total) concentration of payload and *C*_sup_ represents the payload concentration in the supernatant. Values represent the average of 6 independent analyses for 10 mM WGI-0 in MES, pH 7.5, and all other values are based on 5 independent analyses for each system.

### Quantification of Dense-Phase Peptide Concentration

Condensates were prepared as described above. Droplet suspensions were centrifuged at 20 °C and 17K rcf for 30 min. The supernatants were collected and analyzed by absorbance spectroscopy at λ = 280 nm using a Synergy H1 plate reader (BioTek). For quantification, a calibration curve was generated: WGI-0 was dissolved in 1 mM in MES buffer (20 mM, pH 7.5) containing 10 mM CuSO_4_ and serially diluted to final concentrations of 0.02, 0.05, 0.1, 0.2, 0.4, 0.6, and 0.8 mM. Supernatants were further diluted (1:5, 1:10, and 1:20 for 5, 10, and 20 mM) in MES buffer with 10 mM CuSO_4_, and absorbance was recorded in triplicate at 280 nm. Peptide concentrations in the dilute phase were determined using the calibration curve. % of peptides in the dense phase was determined using the following eq 2 and multiplying by the dilution factor.

$$\left(\frac{C_{\mathrm{initial}}-C_{\mathrm{dilute}}}{C_{\mathrm{initial}}}\right)\times 100\%$$ 2

### Product Synthesis and Purification

A mixture of 4-ethynylanisole (100 mg, 109 μL) and AHC (152.4 mg) in DMF (2 mL) was prepared. To this solution, CuSO_4_ (188 mg) dissolved in 1 mL of ddw and NaAsc (223 mg) dissolved in 1 mL of ddw were added sequentially. The reaction was stirred overnight under an argon atmosphere. Upon completion, the reaction mixture was quenched with ddw and extracted three times with ethyl acetate. The organic layer was concentrated under reduced pressure to afford the crude product. Yield: 83%. H^1^ NMR (400 MHz, DMSO): δ 8.89 (s, 1H), 8.63 (s, 1H), 7.88 (d, 2H), 7.76 (d, 1H), 7.05 (d, 2H), 6.91 (d, 1H), 6.88 (s, 1H), 3.81 (s, 3H). LCMS: 336.31 [M + H]^+^.

### Preparation of Reactions in Condensates and HPLC Analysis of Reaction Kinetics

Peptide solutions were prepared as described above. AHC and 4-ethynylanisole (both reactants were dissolved at 10 mM in t-BtOH) were added to peptide solution at a final concentration of 0.5 mM. NaAsc (stock solution 1 M in ddw) was added at a final concentration of 20 mM, and the solutions were incubated for 30 min in argon. Then, CuSO_4_ was added at a final concentration of 10 mM, initiating condensate formation.

To analyze reaction kinetics in HPLC, ACN was added at a final volume percentage of 50% v/v to quench the reaction at the desired time point (0–60 min), and the vials were incubated overnight. Product formation was monitored over time using an analytical reverse-phase HPLC (RP-HPLC, Thermo Fisher) Dionex SD Ultimate 3000 UHPLC standard system equipped with a diode array detection (DAD) detector. Mobile phases were (A) H_2_O (0.1% trifluoroacetic acid; 12) and (B) ACN (0.1% TFA); the stationary phase was a CS chromatography MultiHigh C18 column (250 mm × 4.6 mm, 5 μm particle size, and 100 Å pore size, 5861272), using the gradient 35–40% mobile phase B for 24 min at a flow rate of 1 mL/min. Time 0 of the reaction represents reactions that were quenched immediately after addition of CuSO_4_. We obtained product concentrations by using a calibration curve of the product. For this, the product was dissolved at 1 mM stock solution in ACN and then diluted with ACN to 0.2, 0.4, 0.5, 0.6, and 0.8 mM. All solutions were diluted once more with 5% t-BtOH (%v/v) in ddw (ratio 1:1) to meet the conditions of the kinetic HPLC analysis. For product quantification, we first analyzed the peak area at λ_abs_ = 214 nm as a function of predetermined concentration to create a calibration curve equation using linear regression. Then, we could obtain product concentration for each reaction time point based on the calibration curve. Data points represent averages of at least three independent measurements. The statistical test used to compare the reactions rates was a 2-sided *t* test. All *p* value data are presented in Tables S1–S3 in the SI.

Since the click reaction is a second-order reaction, the reaction rate was calculated based on the following eq 3:

$$\mathrm{rate}=k[C_{\mathrm{azide}}]\times [C_{\mathrm{ethynyl}}]$$ 3

where both [*C*_azide_] and [*C*_ethynyl_] = 500 μM. The reaction constant (*k*) values were calculated from the slope of the kinetic data based on linear regression of the first three time points using the following eq 4:

$$\frac{1}{a-x\left(t\right)}=kt+\frac{1}{a}$$ 4

where *a* is the initial concentration of the reactants (500 μM) and *x* is the product concentration at time *t*.

### Confocal Laser Scanning Microscopy (CLSM) Analysis of Reaction Kinetics

For each condensate system, condensates in either citrate or MES buffer were prepared as described above. 70 μL of the solution was transferred into a vial insert and incubated under Ar for 30 min. Then, 0.7 μL of CuSO_4_ (1 M in ddw) was added to each vial. At the specific time point (10, 20, and 40 min), the whole reaction solution was transferred to a Pluronic F-127-coated 96-well plate. The solution was imaged using a Zeiss LSM 900 inverted confocal microscope, using an λ_ex_ = 405 nm laser, a collection emission range of λ_em_ = 410–700 nm, and an objective 40×/1.2 Imm Korr DIC M27. Images were taken over time, for 40 min. The collected images were taken using the z-stack mode. Condensates of each sample were analyzed using Zen blue 3.2 software (Zeiss) to show the average fluorescence in condensates over time and background fluorescence over time. The numbers of condensates/dilute phase areas analyzed for each system were as follows: *n* = 101, 72, and 94 for 5 mM WGI-1 in citrate buffer *t* = 10, 20, and 40 min, respectively; *n* = 92, 90, and 110 for 5 mM WGI-0 in citrate buffer *t* = 10, 20, and 40 min, respectively; *n* = 51, 60, and 78 for 20 mM WGI-1 in MES buffer *t* = 10, 20, and 40 min, respectively; *n* = 70, 87, and 47 for 20 mM WGI-0 in MES buffer *t* = 10, 20, and 40 min, respectively. For the real-time CLSM analysis of reaction kinetics in air, 5 mM of WGI-0 was dissolved in citrate buffer; subsequently, the reactants and NaAsc were added. Then, 40 μL of the reaction mixture was transferred to a Pluronic F-127-coated 96-well plate, and 10 mM CuSO_4_ was added directly into the well. All images were taken over time using a Zeiss LSM 900 inverted confocal microscope, using an λ_ex_ = 405 nm laser, using an objective 40×/1.2 Imm Korr DIC M27. Each image was analyzed by using Z-stacking. For the CLSM analysis of WGI-2 condensate recruitment of AHC, condensates were prepared as mentioned above using 5 mM WGI-2 in citrate buffer. 10 mM AHC was added to peptide solution or *t*-BuOH in the control sample to maintain the dilution effect. Droplets were formed as the peptide was dissolved. Line profiling was taken to analyze the reactant localization in the dense phase.

### Transmission Electron Microscopy (TEM) Analysis

Samples of WGI-2 condensates were prepared as previously described. 5 μL of the sample solution was applied to an FCF400-Cu grid (FORMVAR/carbon film, 400 mesh copper) and incubated for 2 min. Excess solution was removed by blotting the grid with a filter paper, then washing with 5 μL of ddw, and blotting immediately, followed by staining with 5 μL of 50% v/v uranyless solution for 1 min. After blotting excess stain solution, the grid was air-dried overnight. The negatively stained sample was imaged in a JEM-1400Plus TEM operating at 80 kV. Images were recorded using an SIS Megaview III camera and iTEM, the TEM imaging platform (Olympus).

### Encapsulation Efficiency (EE) Analysis of Rhodamine B Using Absorbance Spectroscopy

Condensates were formed by dissolving each peptide at 10 mM in citrate buffer, pH 5.4, and then adding rhodamine B (10 μM, dissolved in ddw). Subsequently, 10 mM CuSO_4_ was added to initiate LLPS. For samples of WGI-2 without CuSO_4_, ddw was added instead. Droplet solutions were centrifuged at 20 °C and 17K rcf for 30 min. Supernatants were collected from each sample and analyzed using absorbance spectroscopy at λ = 570 nm using a BioTek H1 synergy plate reader. To obtain concentrations of rhodamine B in the supernatants, we used a calibration curve. For this, rhodamine B was dissolved in ddw and serially diluted to final concentrations of 5, 10, 20, 30, 40, 50, 60, 70, 80, and 90 μM. After further dilution of 1:9 in citrate buffer, the absorbance of each sample was measured in triplicates at λ = 570 nm. The %EE values were obtained using the following eq 5:

$$\left(\frac{C_{i}-C_{\sup}}{C_{i}}\right)\times 100$$ 5

where *C*_i_ represents the initial (total) concentration of rhodamine B and *C*_sup_ represents the rhodamine B concentration in the supernatant.

### FRAP Analysis

Fluorescence recovery after photobleaching (FRAP) experiments were performed using a Zeiss 900 LSM confocal microscope by tracking the fluorescent signal of 10 μM rhodamine B or fluorescein as the payload. Condensates were formed by 10 mM of each peptide and 10 mM CuSO_4_ in citrate buffer, pH 5.4. For samples of WGI-2 without CuSO_4_, ddw was added instead to maintain the effect of dilution. For MES systems, 20 mM of each peptide was dissolved in MES buffer, pH 5.7; then, CuSO_4_ was added to a final concentration of 10 mM. All the solutions were imaged using coated wells (96-well plate) by a solution of a Pluronic F-127 surfactant, dissolved in buffer at 5 mg/mL based on a protocol by Rosen and Yao.^37^ Photobleaching was performed at a circular area with a diameter of 1.2 μm using 10 iterations of a λ_ex_ = 488 nm laser for rhodamine B and for fluorescein at 100% intensity, with an objective 40×/1.2 Imm Korr DIC M27, and subsequent recovery of the fluorescence at the bleached area was recorded and analyzed with Zen Blue 3.2 software. Photobleaching correction and recovery times were calculated using OriginLab 9.95. Each experiment was normalized between 0% (bleach intensity) and 100% (intensity before bleaching). Then, the normalized data were fitted to the next equation to extract the fitting parameters: *y* = *a* – *bc* ^*x*^, where *a* is the recovery that was measured. *t*_1/2_ values of WGI-0 and WGI-1 were calculated for each experiment using *a*, *b*, and *c* extracted from the fitting, with the following eq 6:

$$t_{1/2}=\log_{c}\left(\frac{0.5a}{b}\right)$$ 6

The final FRAP recovery curves and the $t_{1/2}$ data are the average of recovery curves collected from *n* = 11 condensates for WGI-0, *n* = 17 for WGI-1, *n* = 8 for WGI-2, *n* = 9 for WGI-2 without CuSO_4_, *n* = 10 for 20 mM WGI-0 in MES, and *n* = 10 for 20 mM in MES.

### Scanning Transmission Electron Microscopy (STEM) and Energy-Dispersive Spectroscopy (EDS) Analysis

Samples were prepared as previously described. 5 or 2 μL of the sample solution was applied to an FCF400-Cu grid (FORMVAR/carbon film, 400 mesh copper) and incubated for 2 min. Excess solution was removed by blotting the grid with a filter paper, then washing with 5 μL of ddw, and blotting immediately. After excess stain solution was blotted, the grid was air-dried overnight. STEM was conducted on a probe-corrected Spectra 200 (S)­TEM, manufactured by Thermo Fisher Scientific, Inc. (USA), equipped with an X-type cold-field emission gun. The STEM was operated at a 80 kV accelerating voltage; the probe current, measured in vacuum, was ∼95 pA; the probe collection semiangle was ∼30°; the camera length was set to 98 mm. The microscope was equipped with a Super-X EDS system that enables it to acquire elemental maps. The pressure reading in the sample region (column) was as low as ∼5 × 10^–6^ Pa (∼3.75 × 10^–8^ Torr). Images were recorded using segmented-BF/DF (Panther, 8 segments) and high-angle annular dark-field (HAADF) imaging (Fischione, USA-OEM) detectors. Data acquisition and processing were conducted using the manufacturer’s software, Velox 3.15.

### Cu^2+^ Localization by ICP-MS and Absorption Spectroscopy

Condensates were formed as previously described (5 mM peptide, 10 mM CuSO_4_, and 20 mM citrate buffer, pH 5.4), and then, droplet solutions were centrifuged at 20 °C, at 17K rcf for 30 min. Supernatants were collected from each sample, Cu^2+^ was measured by ICP-MS (7800, Agilent), and absorbance spectroscopy at λ = 750 nm using a BioTek H1 synergy plate reader. To obtain Cu^2+^ concentrations in the supernatants, we used calibration curves. For this, Cu^2+^ was dissolved in ddw and serially diluted to final concentrations of 5, 6, 8, 9, and 10 mM for absorbance spectroscopy (Figure S16b) and 10.581, 56.705, 199.63, and 986.179 ng/mL for ICP-MS (Figure S16C).

## Supplementary Material



## Data Availability

Additional data supporting the findings presented in main text figures are available on DOI 10.5281/zenodo.17236966.

## Notes

HPLC data of peptides, reaction mechanism schematics, absorbance and fluorescence spectroscopy, bright-field microscopy of condensates, calibration curves for HPLC and absorbance spectroscopy of reactants and products and dyes, TEM images of condensates and peptide aggregation, CLSM images of condensates over the reaction course, NMR spectra of products, STEM EDS, and tables of numerical values of EE and *p* values (PDF)
