# Supplementary material for: Regulation of Chemical Transformation in Designer Peptide Biomolecular Condensates
Source: ACS Appl Mater Interfaces. 2026 Feb 18;18(8):12432–41. doi: 10.1021/acsami.5c21674 (PMC12964334; doi:10.1021/acsami.5c21674)
Supplement: Supplementary file 1 [file am5c21674_si_001.pdf]

Supporting Information

Regulation of Chemical Transformation in Designer Peptide Biomolecular Condensates

*Shirel Veretnik, Avigail Baruch Leshem and Ayala Lampel**

Shirel Veretnik, Avigail Baruch Leshem

Shmunis School of Biomedicine and Cancer Research, George S. Wise Faculty of Life Sciences, Tel Aviv University 69978, Israel.

Ayala Lampel

Shmunis School of Biomedicine and Cancer Research, George S. Wise Faculty of Life Sciences, Tel Aviv University, Israel;

Center for Nanoscience and Nanotechnology Tel Aviv University, Tel Aviv 69978, Israel;

Center for the Physics and Chemistry of Living Systems Tel Aviv University, Tel Aviv 69978, Israel;

Leibniz Institute of Polymer Research Dresden Max Bergmann Center of Biomaterials Dresden 01069 Dresden, Germany.

Corresponding author E-mail: ayalalampel@tauex.tau.ac.il

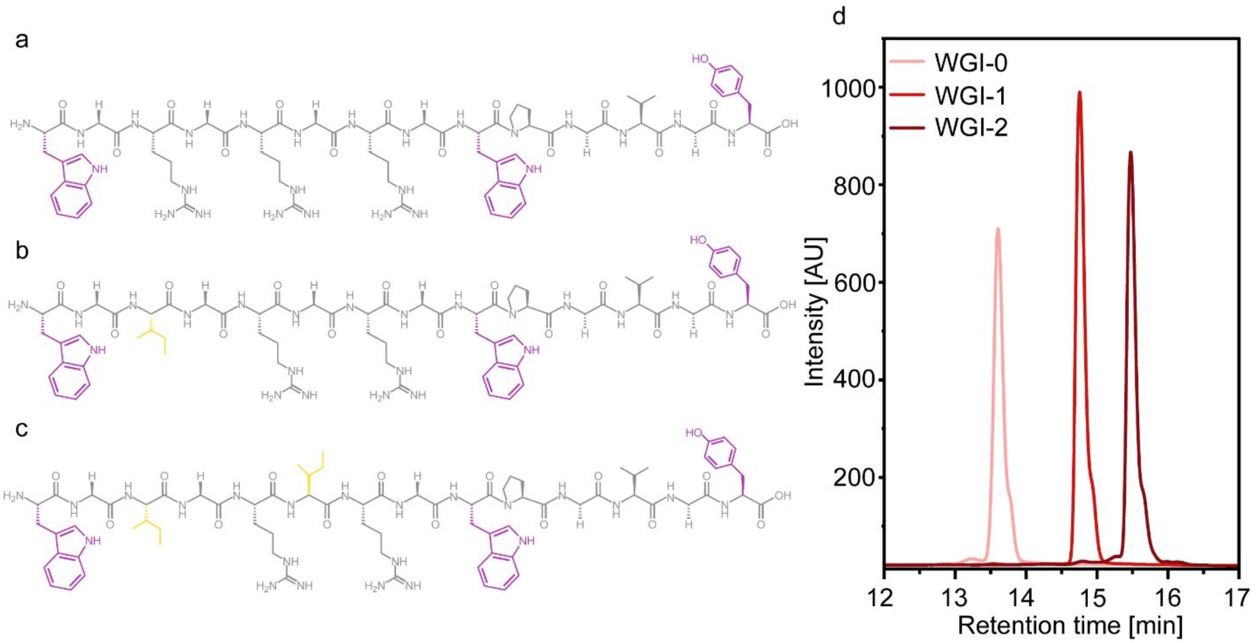

Figure S1. a-c. Chemical structures of (a) WGI-0, (b) WGI-1, and (c) WGI-2 peptides. **d.** HPLC chromatograms showing the retention time of WGI-0, WGI-1 and WGI-2.

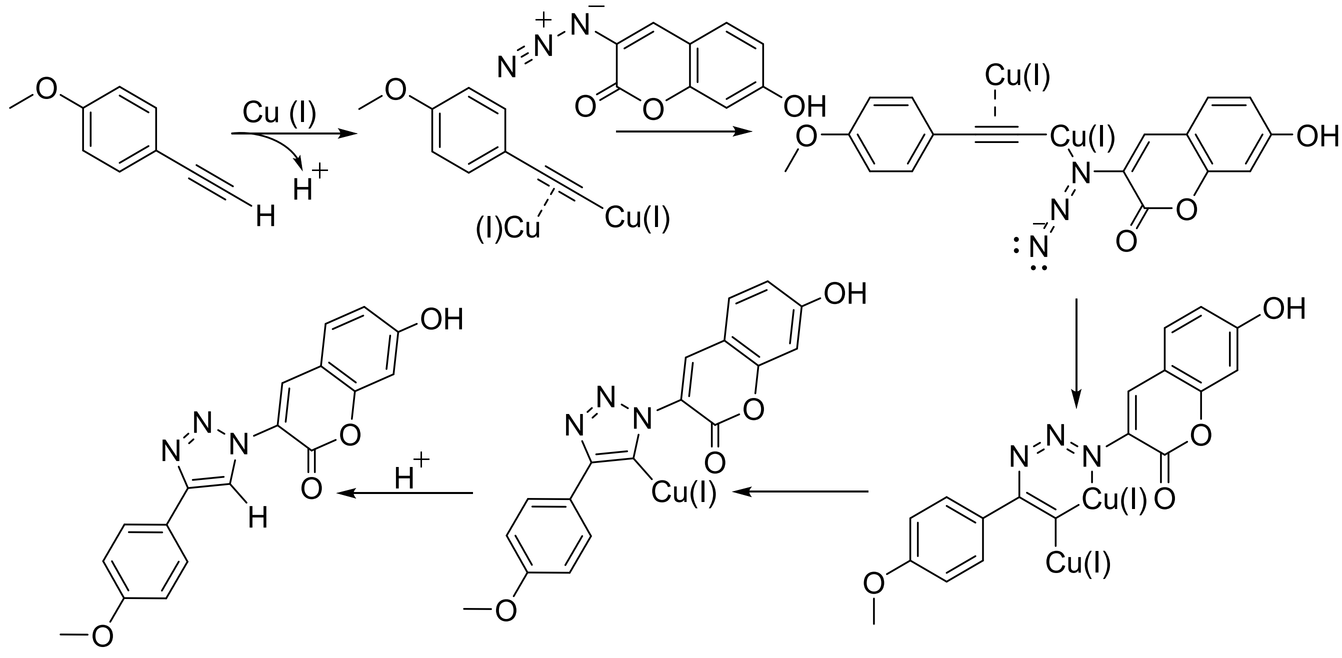

Figure S2. Click reaction mechanism between 3-Azido-7-hydroxycoumarin (AHC) and 4-ethynylanisole to form 1,4-bis(4-methoxyphenyl)-1H-1,2,3-triazole.

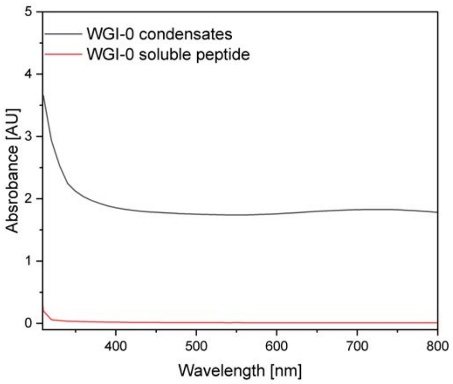

Figure S3. Absorbance spectroscopy analysis of WGI-0 condensates (5 mM in citrate buffer pH 5.5 with 10 mM $CuSO_4$) compared to soluble WGI-0 peptide (5 mM in citrate buffer in the absence of $CuSO_4$).

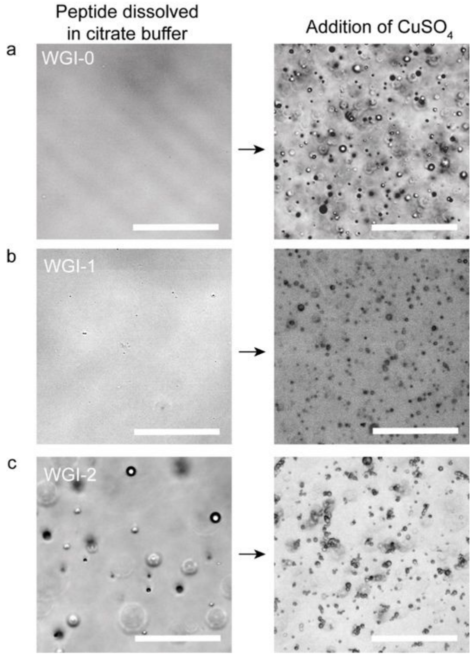

Figure S4. Droplets formation by the addition of $CuSO_4$ to a 5 mM of **a.** WGI-0, **b.** WGI-1, and **c.** WGI-2. Peptides were dissolved in citrate buffer at pH 5.5, then $CuSO_4$ was added to the peptide solutions, resulting in condensate formation. Scale bar=50 µm.

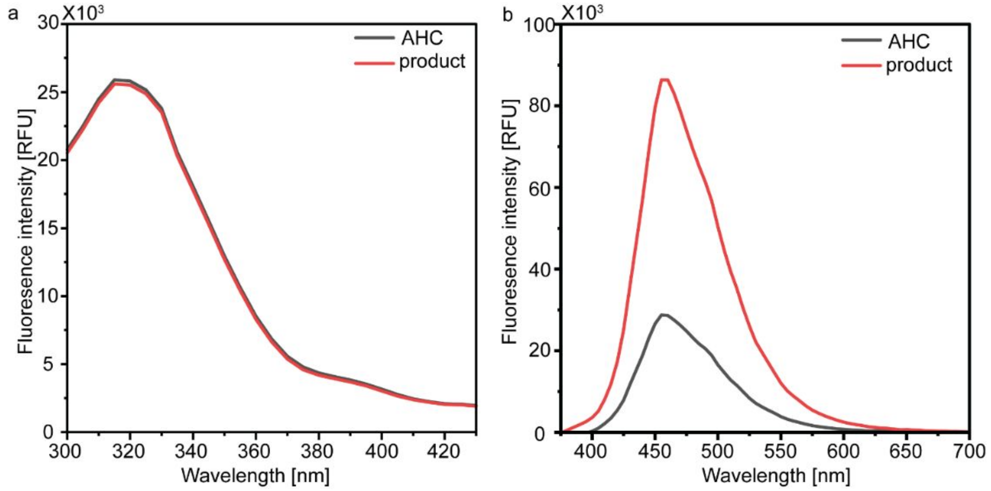

Figure S5. a. Excitation spectra of product and AHC showing λ_{ex}=330 nm. **b**. Emission spectra of product and AHC showing λ_{em}=460 nm.

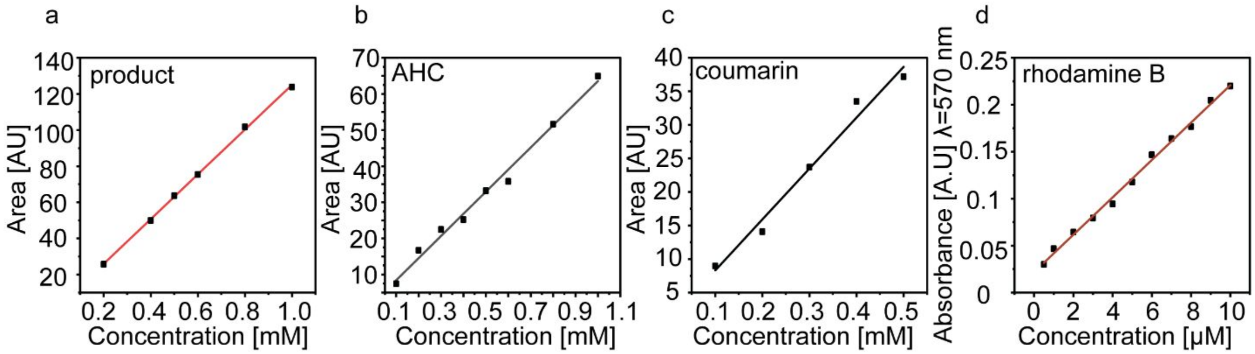

Figure S6. Calibration curves of the (**a**) triazole ring product, **(b)** the reactant AHC and (**c**) coumarin analyzed by HPLC. **d**. Rhodamine B calibration curve analyzed by absorbance spectroscopy at λ=570nm.

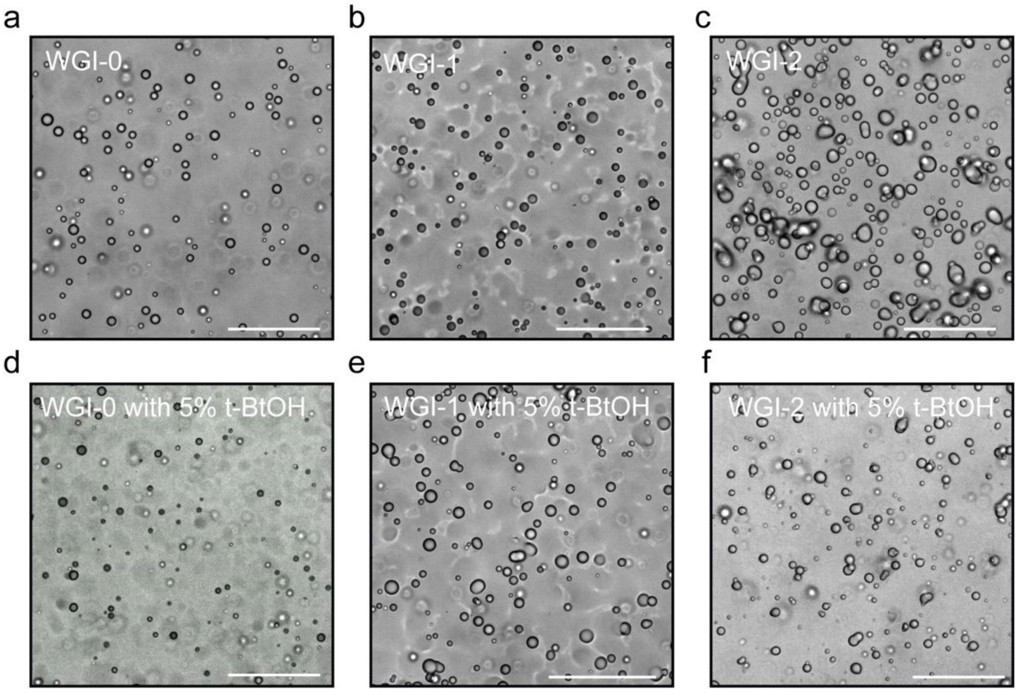

Figure S7. Brightfield microscopy analysis of peptide condensates in the absence or presence of 5% t-BtOH. Peptide condensates using 5 mM of each peptide in citrate buffer pH 5.5. Scale bar=25 μm.

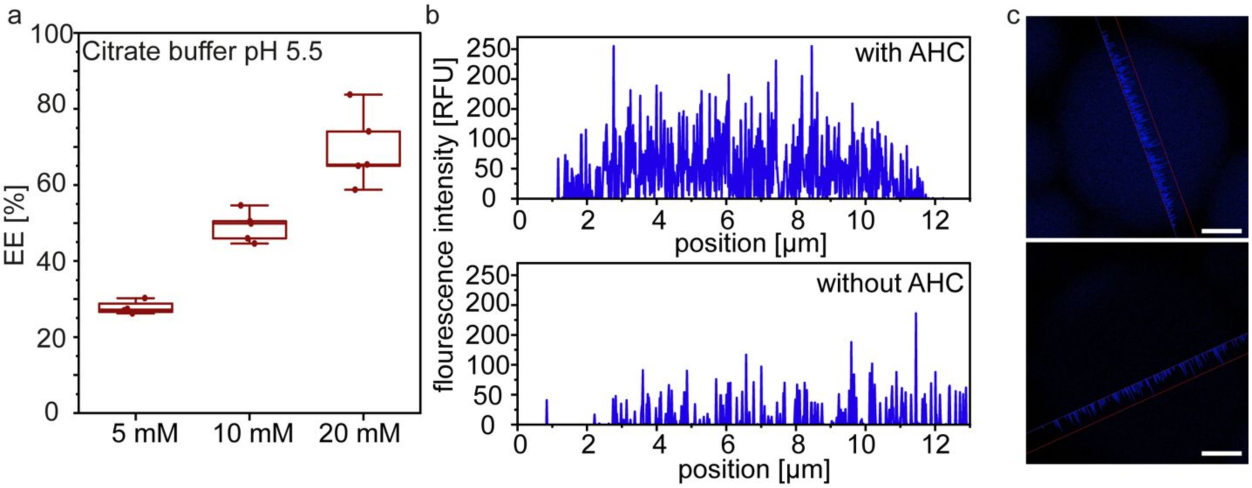

Figure S8. Reactant recruitment to WGI-2 condensates. (**a**) Encapsulation efficiency (EE) of AHC in 5 mM, 10 mM and 20 mM WGI-2 condensates formed in citrate buffer pH 5.4 with 10 mM $CuSO_4$. (**b**) CLSM line-scan analysis of WGI-2 (with $CuSO_4$) droplets with and without AHC and (**c**) droplets images accordingly. Scale bars=2 μm.

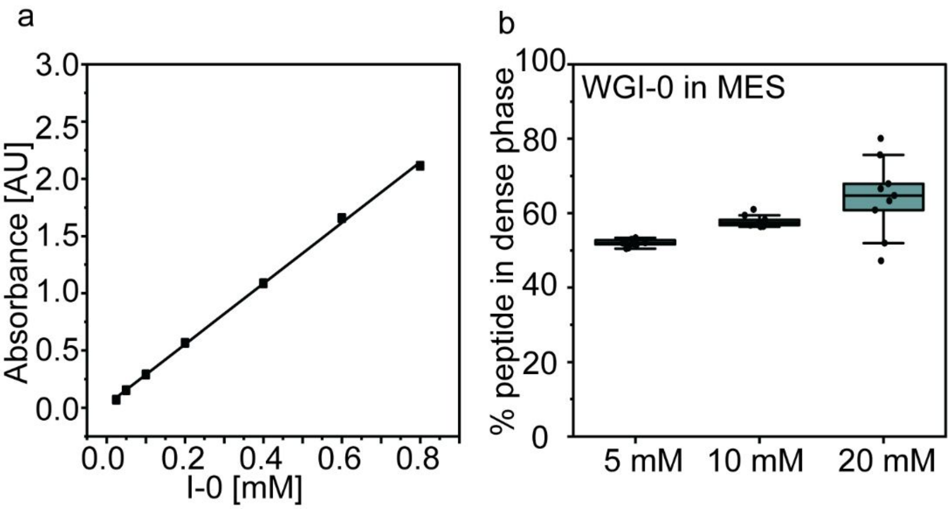

Figure S9. a. Absorbance spectroscopy calibration curve of WGI-0. b. Quantification of WGI-0 dense-phase concentration at increasing total peptide concentration, analyzed by absorbance spectroscopy.

Table S1. Summary of AHC encapsulation efficiency (EE) in WGI-0 and WGI-1 condensates in citrate buffer (pH=5.4) or MES buffer (pH=5.7).

Peptide	Conc. [mM]	buffer	EE%	Peptide	Conc. [mM]	buffer	EE%
WGI-0	5	citrate	7.2±5.5	WGI-1	5	citrate	35.8±3.1
WGI-0	10	citrate	26.3±15.5	WGI-1	10	citrate	45.7±2.3
WGI-0	20	citrate	43.0±6.7	WGI-1	20	citrate	65.1±7.5
WGI-0	5	MES	12.2±3.1	WGI-1	5	MES	66.5±8.8
WGI-0	10	MES	48.8±4.8	WGI-1	10	MES	81.6±3.8
WGI-0	20	MES	63.7±2.6	WGI-1	20	MES	91.6±1.6

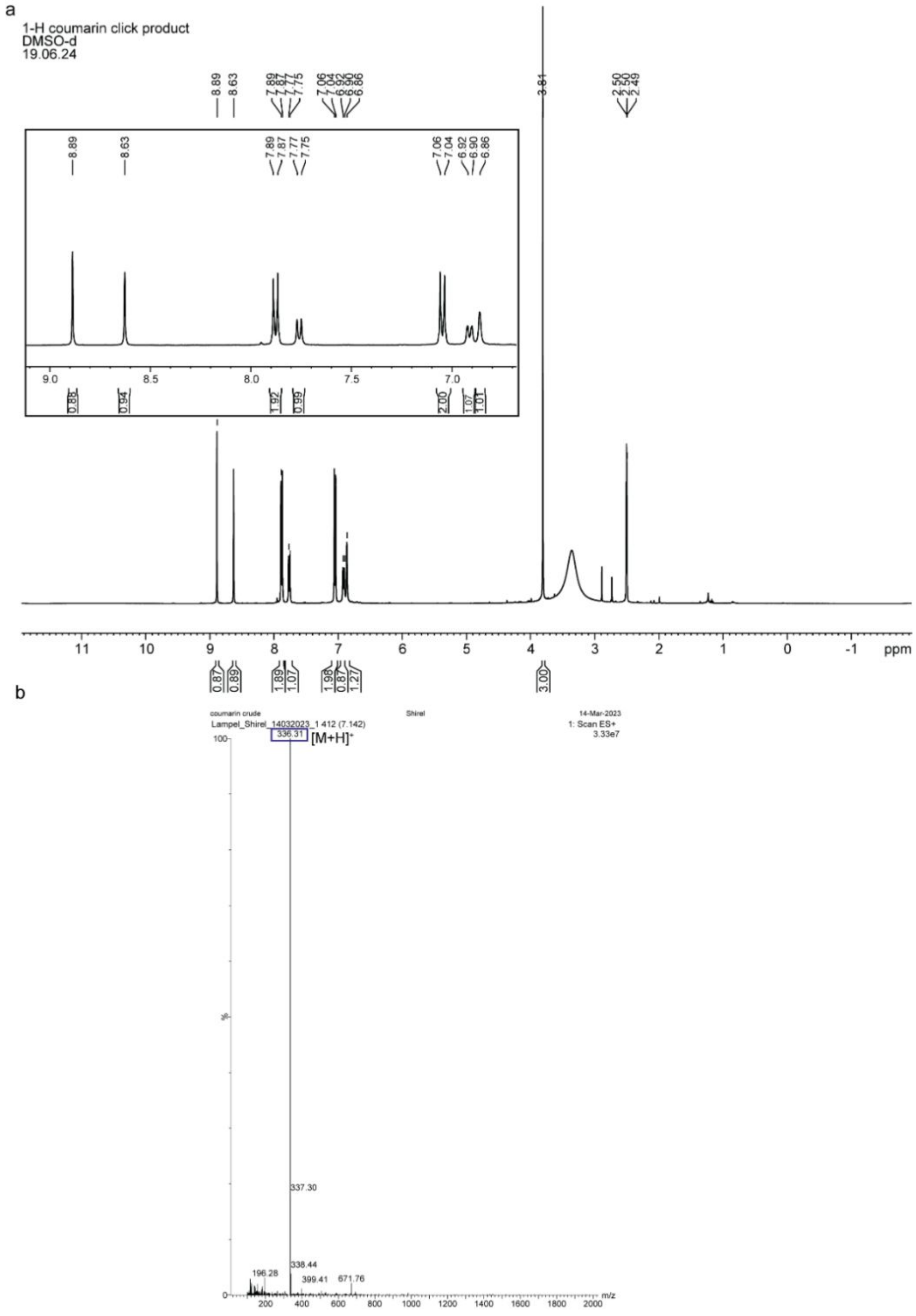

Figure S10. a. H[1] NMR spectrum and **b.** MS spectrum of 7-hydroxy-3-(4-(4-methoxyphenyl)-1H-1,2,3-triazol-1-yl)-2H-chromen-2-one.

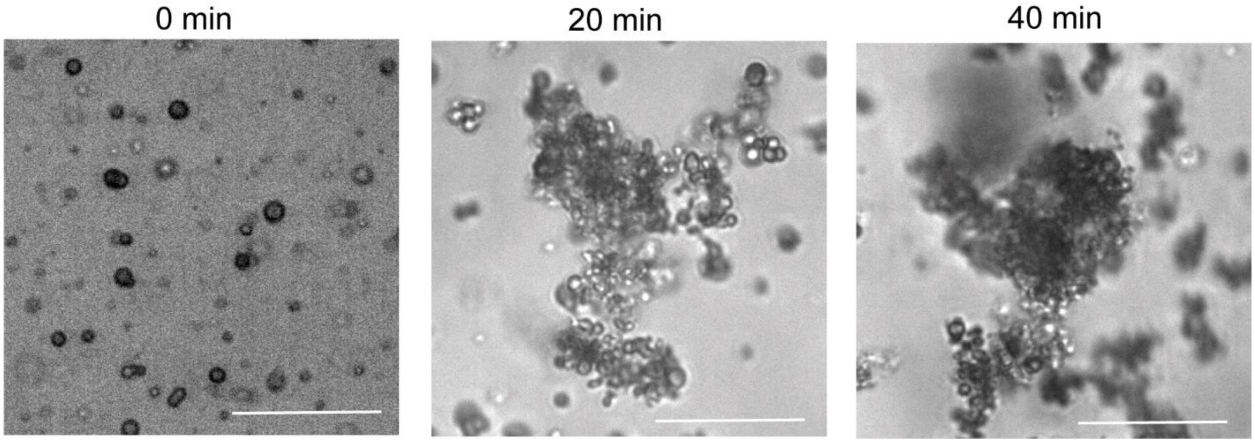

Figure S11. Condensates formed by 5 mM WGI-1 in citrate buffer, pH 5.5 with 1 mM reactants, 20 mM NaASc, and 10 mM CuSO4, at t=0 min, 20 min, and 40 min. Scale bar=50 µm.

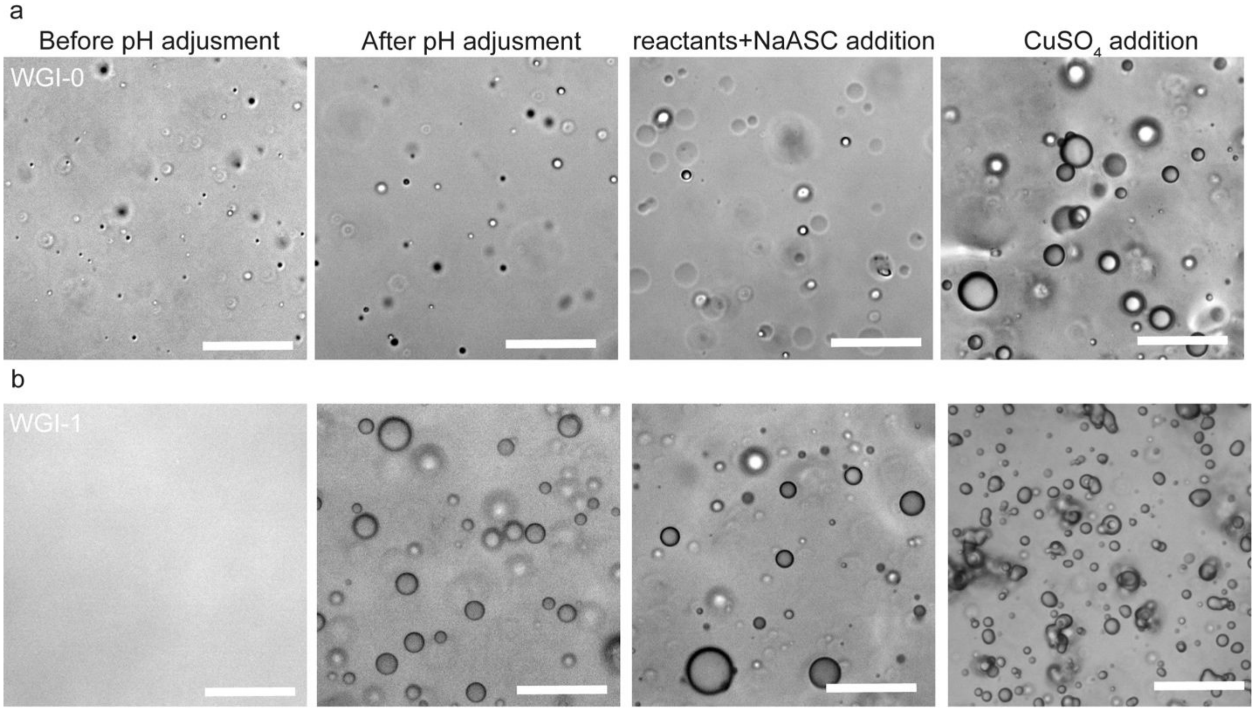

Figure S12. Order of sample preparation of reactions in MES buffer systems. **a**. i. Peptides were dissolved in MES buffer, ii. and their pH was adjusted to 7.5, resulting condensates formation. iii. Reactants and NaAsc were added to the peptide solutions and incubated for 30 minutes under Ar. iv. CuSO4 was added to initiate the reaction. v. reaction occurring vi. The reaction was quenched following ACN addition. vi. HPLC analysis of the sample. Images of (**b**) WGI-0 and (**c**) WGI-1 (left to right) before pH was adjust and afterwards; following the reactants and NaAsc addition and following $CuSO_4$ addition. Scale bar=25 μm.

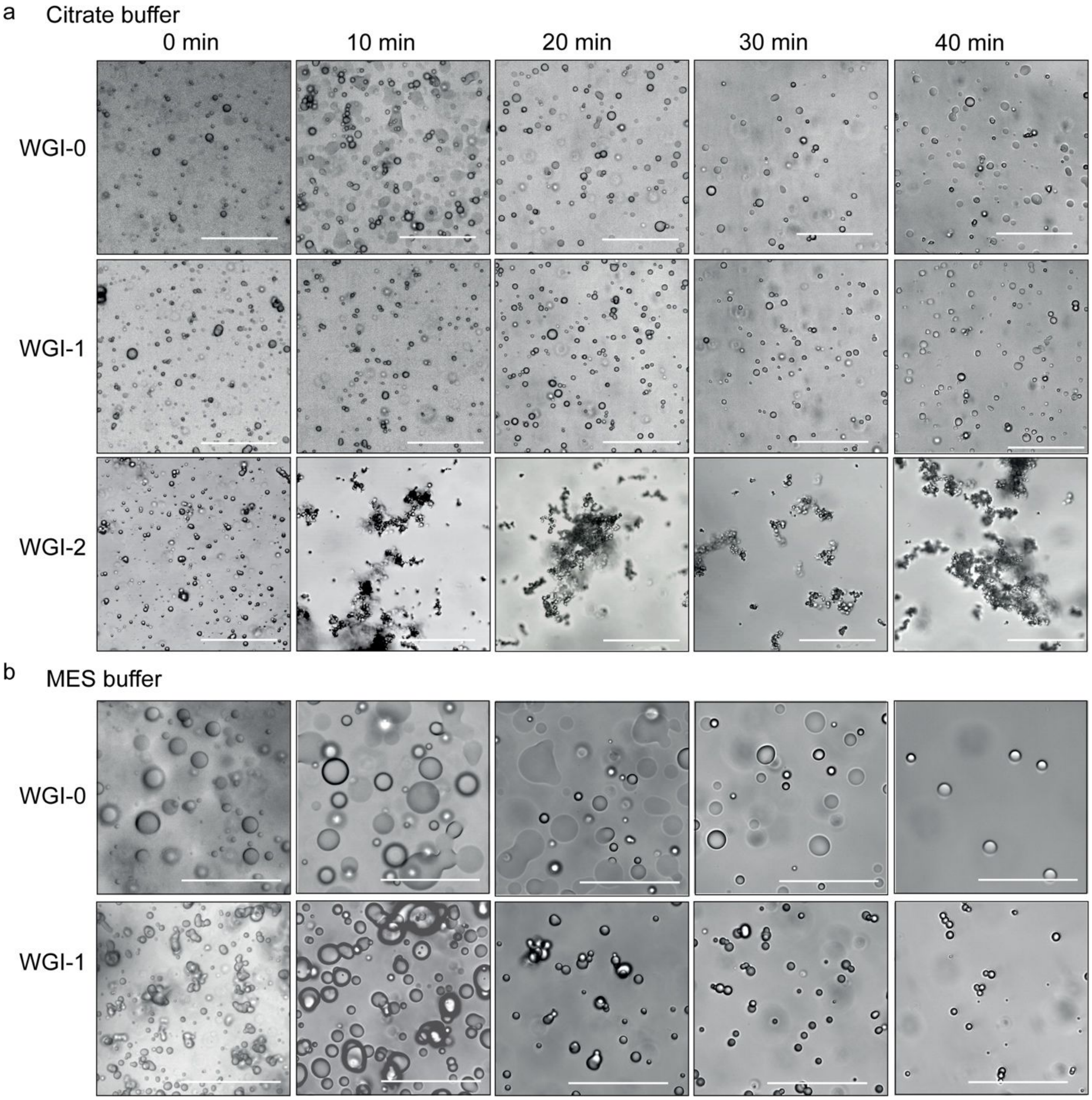

Figure S13. Brightfield microscopy analysis of condensates over reaction time in (**a**) citrate buffer (5 mM peptide, pH-5.5) and (**b**) MES buffer (20 mM peptide, pH=7.5). Scale bars=25 μm.

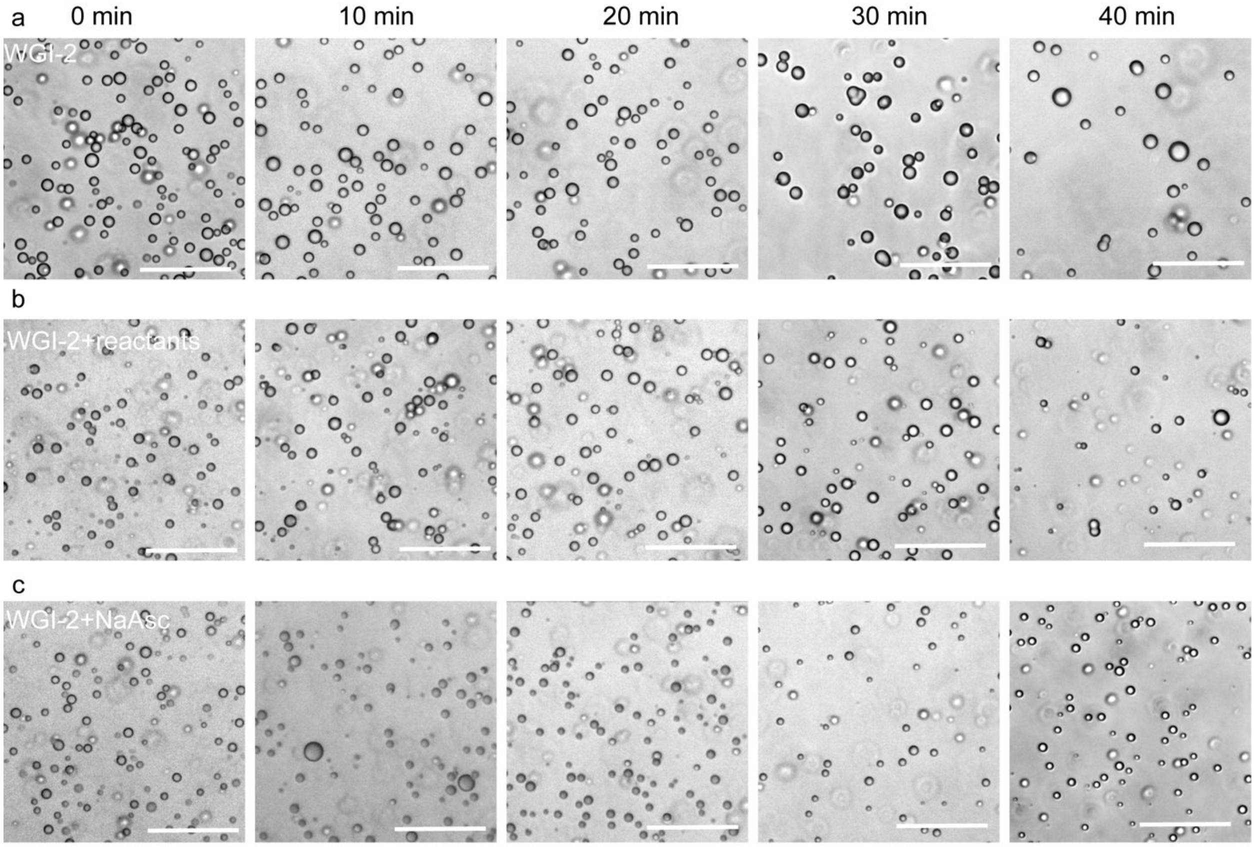

Figure S14. Microscopy analysis of (a) 5mM WGI-2 condensate morphology in the presence of (b) reactants without NaAsc and (**b**) NaAsc without the reactants over time. Scale bar= 25 μm.

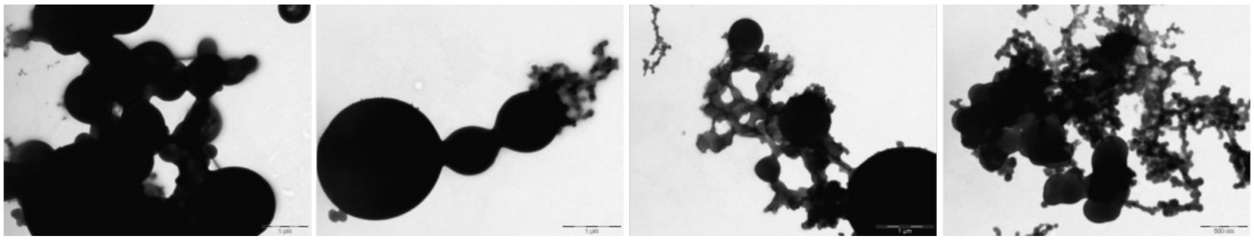

Figure S15. Transmission Electron microscopy (TEM) images of WGI-2 condensates (5 mM) formed with 10 mM $CuSO_4$ in the presence of reactants and NaAsc after 40 minutes of reaction under Ar.

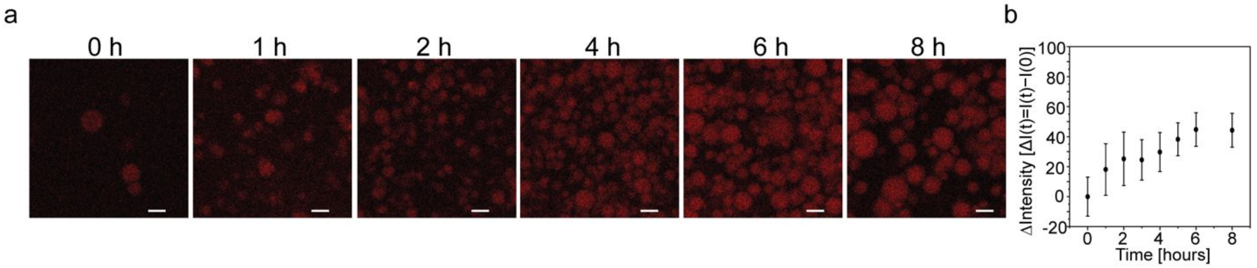

Figure S16. Real-time CLSM analysis of reaction kinetics in WGI-0 in citrate buffer (5 mM peptide, pH=5.5) showing product fluorescence intensity (λ_{ex}=405 nm). **a**. CLSM images. **b**. Relative change in product fluorescence intensity over time obtained from the CLSM imaging. Values represent averages of n=38, 83, 77, 93, 93, 115, 114, 96 for 0 h, 1 h, 2 h, 3 h, 4 h, 5 h, 6 h, 8 h. Error bars represent S.D. Scale bar=2 μm.

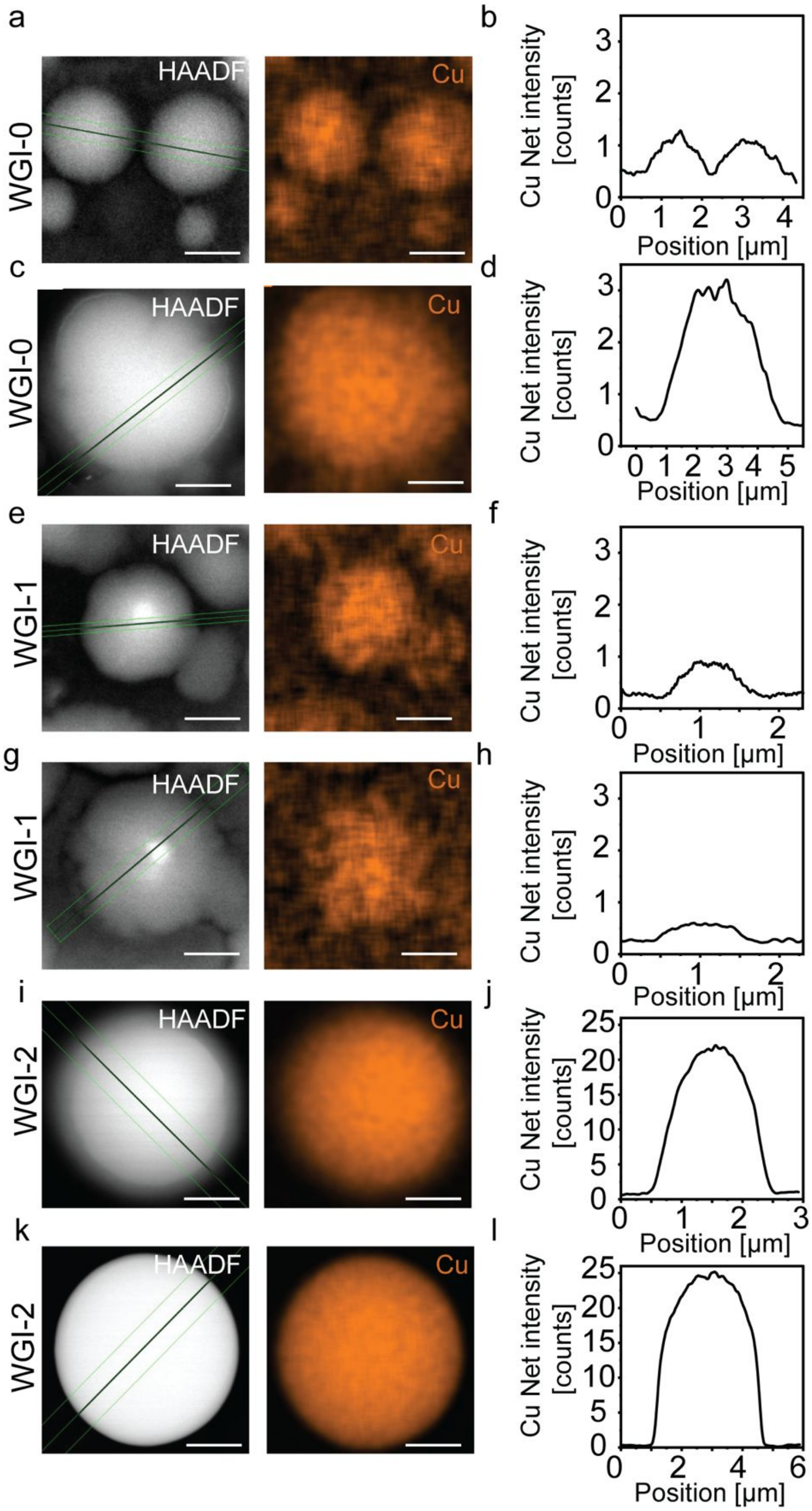

Figure S17. Scanning transmission electron microscopy (STEM) energy dispersive spectroscopy (EDS) analysis of WGI-0 (**a-d**), WGI-1 (**e-h**), and WGI-2 (**i-l**). Left panel: Micrographs showing dark field and

EDS elemental maps of copper (Cu). Right panel: Line scan mapping showing the net intensity of Cu. Scale bar=500 nm

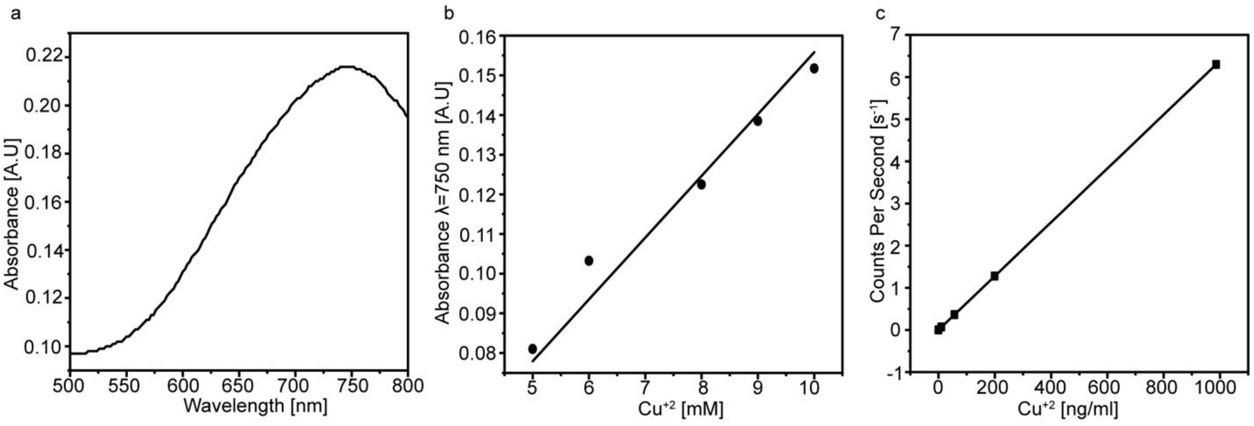

Figure S18. a. Cu^{2+} absorption spectra and (**b-c**) calibration curves of Cu^{2+} in citrate buffer (pH=5.5) using (**b**) absorption spectroscopy and (**c**) ICP-MS.

Table S2: *p*-values of Figure 3e.

	p-value	<0.05 (*)	<0.01 (**)
citrate VS ddw	0.010231	TRUE	FALSE
citrate VS 5mM WGI-0	0.006925	TRUE	TRUE
citrate VS 5mM WGI-1	0.003096	TRUE	TRUE
citrate VS 5mM WGI-2	0.0172	TRUE	FALSE
ddw VS 5 mM WGI-0	0.028013	TRUE	FALSE
ddw VS 5 mM WGI-1	0.010348	TRUE	FALSE
ddw VS 5 mM WGI-2	0.033087	TRUE	FALSE
5 mM WGI-0 VS 5 mM WGI-1	0.25383	FALSE	FALSE
5 mM WGI-0 VS 5 mM WGI-2	0.361099	FALSE	FALSE

Table S3: *p*-values of Figure 3f.

	p-value	<0.05 (*)
WGI-0 VS WGI-1	0.506960297	FALSE
WGI-0 vS buffer	0.239454199	FALSE
WGI-1 VS buffer	0.198867338	FALSE

Table S4: *p*-values of Figure 3g.

	p-value	<0.05 (*)	<0.01 (**)
MES buffer VS ddw	0.119229	FALSE	FALSE
MES buffer VS 20 mM WGI-0	0.03108	TRUE	FALSE
MES buffer VS 20 mM WGI-1	0.010615	TRUE	FALSE
ddw VS 20 mM WGI-0	0.025597	TRUE	FALSE
ddw VS 20 mM WGI-1	0.003481	TRUE	TRUE
20 mM WGI-0 VS 20 mM WGI-1	0.051093	FALSE	FALSE
